# Supplementary figures and images for: Wave-wise comparative genomic study for revealing the complete scenario and dynamic nature of COVID-19 pandemic in Bangladesh
Source: PLoS One. 2021 Sep 29;16(9):e0258019. doi: 10.1371/journal.pone.0258019 (PMC8480844; doi:10.1371/journal.pone.0258019)

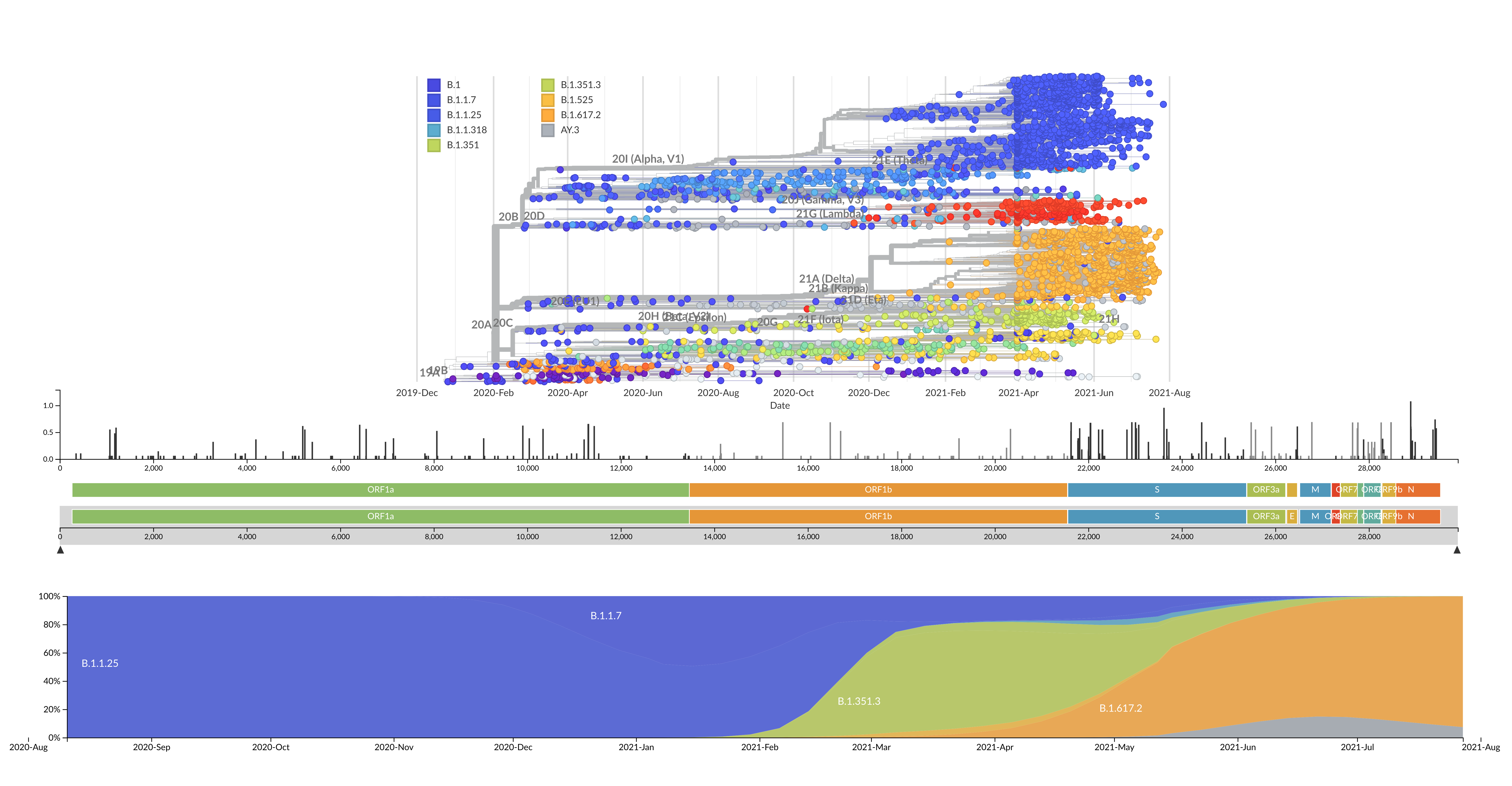

Supplement: S1 Fig — Molecular phylogeny of SARS-CoV-2 genomes from Bangladesh. (PNG) [file pone.0258019.s011.png]
