# Supplementary material for: Wave-wise comparative genomic study for revealing the complete scenario and dynamic nature of COVID-19 pandemic in Bangladesh
Source: PLoS One. 2021 Sep 29;16(9):e0258019. doi: 10.1371/journal.pone.0258019 (PMC8480844; doi:10.1371/journal.pone.0258019)
Supplement: Attachment — Submitted filename: PONE-D-21-19985 (reviewed).pdf [file pone.0258019.s013.pdf]

PLOS ONE

Comparative Genomic Study for Revealing the Complete Scenario of COVID-19 Pandemic in Bangladesh

--Manuscript Draft--

Manuscript Number:	PONE-D-21-19985
Article Type:	Research Article
Full Title:	Comparative Genomic Study for Revealing the Complete Scenario of COVID-19 Pandemic in Bangladesh
Short Title:	Comparative Genomic Study for Revealing the Complete Scenario of COVID-19 Pandemic in Bangladesh
Corresponding Author:	Ishtiaque Ahammad<br>Bioinformatics Division, National Institute of Biotechnology, Ganakbari, Ashulia, Savar, Dhaka-1349, Bangladesh.<br>Dhaka, BANGLADESH
Keywords:	SARS-CoV-2; COVID-19; Comparative Genomics; Molecular Dynamics Simulation; Bangladesh
Abstract:	As the COVID-19 pandemic continues to ravage across the globe and take millions of lives and like many parts of the world, the second wave of the pandemic hit Bangladesh, this study aimed at understanding its causative agent, SARS-CoV-2 at the genomic and proteomic level and provide precious insights about the pathogenesis, evolution, strengths and weaknesses of the virus. As of Mid-June 2021, over 1500 SARS-CoV-2 genomes have been sequenced across the country. From our analyses, it was discovered that the wave-2 samples had a significantly greater average rate of mutation/sample (30.79%) than the wave-1 samples (12.32%). Wave-2 samples also had a higher frequency of deletion, and transversion events. During the first wave, the GR clade was the most predominant but it was replaced by the GH clade in the latter wave. The B.1.1.25 variant showed the highest frequency in wave-1 while in case of wave-2, the B.1.351.3 variant, was the most common one. A notable presence of the delta variant, which is currently at the center of concern, was also observed. Comparison of the Spike protein found in the reference and the 3 most common lineages found in Bangladesh namely, B.1.1.7, B.1.351, B.1.617 in terms of their ability to form stable complexes with ACE2 receptor revealed that B.1.617 had the potential to be more transmissible than others. Importantly, no indigenous variants have been detected so far which implies that the successful prevention of import of foreign variants can diminish the outbreak in the country.
Order of Authors:	Ishtiaque Ahammad
	Mohammad Uzzal Hossain
	Anisur Rahman
	Zeshan Mahmud Chowdhury
	Arittra Bhattacharjee
	Keshob Chandra Das
	Chaman Ara Keya
	Md. Salimullah
Opposed Reviewers:	
Additional Information:	
Question	Response
**Financial Disclosure**<br><br>Enter a financial disclosure statement that	The author(s) received no specific funding for this work.

Powered by Editorial Manager® and ProduXion Manager® from Aries Systems Corporation

describes the sources of funding for the work included in this submission. Review the submission guidelines for detailed requirements. View published research articles from *PLOS ONE* for specific examples.<br><br>This statement is required for submission and **will appear in the published article** if the submission is accepted. Please make sure it is accurate.<br><br>**Unfunded studies**<br>Enter: *The author(s) received no specific funding for this work.*<br><br>**Funded studies**<br>Enter a statement with the following details:<br>• Initials of the authors who received each award<br>• Grant numbers awarded to each author<br>• The full name of each funder<br>• URL of each funder website<br>• Did the sponsors or funders play any role in the study design, data collection and analysis, decision to publish, or preparation of the manuscript?<br>• **NO** - Include this sentence at the end of your statement: *The funders had no role in study design, data collection and analysis, decision to publish, or preparation of the manuscript.*<br>• **YES** - Specify the role(s) played.<br><br>* typeset	
**Competing Interests**<br><br>Use the instructions below to enter a competing interest statement for this submission. On behalf of all authors, disclose any competing interests that could be perceived to bias this work—acknowledging all financial support and any other relevant financial or non-financial competing interests.<br><br>This statement is required for submission and **will appear in the published article** if the submission is accepted. Please make sure it is accurate and that any funding	The authors have declared that no competing interests exist.

sources listed in your Funding Information later in the submission form are also declared in your Financial Disclosure statement.<br><br>View published research articles from *PLOS ONE* for specific examples.<br><br>**NO authors have competing interests**<br><br>Enter: *The authors have declared that no competing interests exist.*<br><br>**Authors with competing interests**<br><br>Enter competing interest details beginning with this statement:<br><br>*I have read the journal's policy and the authors of this manuscript have the following competing interests: [insert competing interests here]*<br><br>* typeset	
**Ethics Statement**<br><br>Enter an ethics statement for this submission. This statement is required if the study involved:<br><br>• Human participants<br>• Human specimens or tissue<br>• Vertebrate animals or cephalopods<br>• Vertebrate embryos or tissues<br>• Field research<br><br>Write "N/A" if the submission does not require an ethics statement.<br><br>General guidance is provided below. Consult the submission guidelines for detailed instructions. **Make sure that all information entered here is included in the Methods section of the manuscript.**	N/A

**Format for specific study types**<br><br>**Human Subject Research (involving human participants and/or tissue)**<br>• Give the name of the institutional review board or ethics committee that approved the study<br>• Include the approval number and/or a statement indicating approval of this research<br>• Indicate the form of consent obtained (written/oral) or the reason that consent was not obtained (e.g. the data were analyzed anonymously)<br><br>**Animal Research (involving vertebrate animals, embryos or tissues)**<br>• Provide the name of the Institutional Animal Care and Use Committee (IACUC) or other relevant ethics board that reviewed the study protocol, and indicate whether they approved this research or granted a formal waiver of ethical approval<br>• Include an approval number if one was obtained<br>• If the study involved *non-human primates*, add *additional details* about animal welfare and steps taken to ameliorate suffering<br>• If anesthesia, euthanasia, or any kind of animal sacrifice is part of the study, include briefly which substances and/or methods were applied<br><br>**Field Research**<br><br>Include the following details if this study involves the collection of plant, animal, or other materials from a natural setting:<br>• Field permit number<br>• Name of the institution or relevant body that granted permission	
**Data Availability**<br><br>Authors are required to make all data underlying the findings described fully available, without restriction, and from the time of publication. PLOS allows rare exceptions to address legal and ethical concerns. See the PLOS Data Policy and FAQ for detailed information.	Yes - all data are fully available without restriction

A Data Availability Statement describing where the data can be found is required at submission. Your answers to this question constitute the Data Availability Statement and **will be published in the article**, if accepted.<br><br>**Important:** Stating 'data available on request from the author' is not sufficient. If your data are only available upon request, select 'No' for the first question and explain your exceptional situation in the text box.<br><br>Do the authors confirm that all data underlying the findings described in their manuscript are fully available without restriction?	
**Describe where the data may be found in full sentences. If you are copying our sample text, replace any instances of XXX with the appropriate details.**<br><br>• If the data are **held or will be held in a public repository**, include URLs, accession numbers or DOIs. If this information will only be available after acceptance, indicate this by ticking the box below. For example: *All XXX files are available from the XXX database (accession number(s) XXX, XXX.)*.<br>• If the data are all contained **within the manuscript and/or Supporting Information files**, enter the following: *All relevant data are within the manuscript and its Supporting Information files.*<br>• If neither of these applies but you are able to provide **details of access elsewhere**, with or without limitations, please do so. For example:<br><br>*Data cannot be shared publicly because of [XXX]. Data are available from the XXX Institutional Data Access / Ethics Committee (contact via XXX) for researchers who meet the criteria for access to confidential data.*<br><br>*The data underlying the results presented in the study are available from (include the name of the third party*	All relevant data are within the manuscript and its Supporting Information files.

Powered by Editorial Manager® and ProduXion Manager® from Aries Systems Corporation

and contact information or URL).<br>• This text is appropriate if the data are owned by a third party and authors do not have permission to share the data.<br><br>* typeset	
Additional data availability information:	Tick here if the URLs/accession numbers/DOIs will be available only after acceptance of the manuscript for publication so that we can ensure their inclusion before publication.; Tick here if your circumstances are not covered by the questions above and you need the journal's help to make your data available.

Powered by Editorial Manager® and ProduXion Manager® from Aries Systems Corporation

Cover Letter

18/06/2021

To

Editor-in-chief

PLoS ONE

Dear Sir

We are submitting an original research article entitled “Comparative genomic study for revealing the complete scenario of COVID-19 pandemic in Bangladesh” for consideration by PLoS ONE.

The current study revealed significant genomic and proteomic differences in the SARS-CoV-2 viral isolates circulating within the perimeters of Bangladesh between the first and the second wave of the COVID-19 pandemic. We have found out that they differ in terms of distribution of clades, mutations, variants, rate of mutations, and even in terms of their interactions with the host ACE2 receptor. We have also found evidence that the B.1.617 lineage of the virus is likely to be more infectious than others. Notably, any existence of a domestic variant is yet to be detected. A comparative genomic study of such kind can significantly help the scientific community gain insights more about the dynamic evolution of SARS-CoV-2 through waves of the COVID-19 pandemic and its implications in the pathogenicity of the virus.

We confirm that this work is original and has not been published elsewhere, nor is it currently under consideration for publication elsewhere. All authors significantly contributed to the work. All authors read and approved the manuscript. None of the authors have any conflicts of interest to disclose.

Kind regards,

Ishtiaque Ahammad

Scientific Officer

Bioinformatics Division, National Institute of Biotechnology

Ganakbari, Ashulia, Savar, Dhaka-1349, Bangladesh.

Email: ishtiaque.ahammad@northsouth.edu, ishtiaquebioinfo@nib.gov.bd

Manuscript Click here to access/download;Manuscript;Manuscript.docx

Comparative genomic study for revealing the complete scenario of COVID-19 pandemic in Bangladesh

Ishtiaque Ahammad[1#], Mohammad Uzzal Hossain[1#], Anisur Rahman[2#], Zeshan Mahmud Chowdhury[3], Arittra Bhattacharjee[1], Keshob Chandra Das[4], Chaman Ara Keya[3], Md. Salimullah[4*]

[1]*Bioinformatics Division, National Institute of Biotechnology, Ganakbari, Ashulia, Savar, Dhaka-1349, Bangladesh*

[2]*Department of Biotechnology and Genetic Engineering, Noakhali Science and Technology University, Sonapur, Noakhali-3814, Bangladesh*

[3]*Department of Biochemistry and Microbiology, North South University, Bashundhara, Dhaka-1229, Bangladesh*

[4]*Molecular Biotechnology Division, National Institute of Biotechnology, Ganakbari, Ashulia, Savar, Dhaka-1349, Bangladesh*

***Corresponding Author:**

Dr. Md. Salimullah

Chief Scientific Officer

Molecular Biotechnology Division

National Institute of Biotechnology

Ganakbari, Ashulia, Savar, Dhaka-1349, Bangladesh

Tel: 880-2-7788443

E-mail: salim2969@gmail.com

#These authors contributed equally to this work

Abstract

As the COVID-19 pandemic continues to ravage across the globe and take millions of lives and like many parts of the world, the second wave of the pandemic hit Bangladesh, this study aimed at understanding its causative agent, SARS-CoV-2 at the genomic and proteomic level and provide precious insights about the pathogenesis, evolution, strengths and weaknesses of the virus. As of Mid-June 2021, over 1500 SARS-CoV-2 genomes have been sequenced across the country. From our analyses, it was discovered that the wave-2 samples had a significantly greater average rate of mutation/sample (30.79%) than the wave-1 samples (12.32%). Wave-2 samples also had a higher frequency of deletion, and transversion events. During the first wave, the GR clade was the most predominant but it was replaced by the GH clade in the latter wave. The B.1.1.25 variant showed the highest frequency in wave-1 while in case of wave-2, the B.1.351.3 variant, was the most common one. A notable presence of the delta variant, which is currently at the center of concern, was also observed. Comparison of the Spike protein found in the reference and the 3 most common lineages found in Bangladesh namely, B.1.1.7, B.1.351, B.1.617 in terms of their ability to form stable complexes with ACE2 receptor revealed that B.1.617 had the potential to be more transmissible than others. Importantly, no indigenous variants have been detected so far which implies that the successful prevention of import of foreign variants can diminish the outbreak in the country.

Keywords

SARS-CoV-2; COVID-19; Comparative Genomics; Molecular Dynamics Simulation; Bangladesh

1. Introduction

Severe Acute Respiratory Syndrome Coronavirus-2 (SARS-CoV-2), the causative agent of Coronavirus Disease-2019 (COVID-19), has already infected > 177,000,000 people and caused >3,800,000 deaths till mid-June, 2021) (1). Since the influenza outbreak of 1918 COVID-19 is the biggest pandemic of zoonotic origin that we are facing at a global scale (2). The first wave of the pandemic has passed and subsequent waves have already started in many countries (3–7). Insights regarding the transmission and evolution of the virus during these waves are essential to break the chain of infections (8,9). Genomic data can provide some of these crucial insights which can help make pragmatic public health policies (10,11). Besides, genomic surveillance can deliver a deep understanding of the virus' mechanism of survival and reduce fatality during new waves of infection (11–14)

The onset of SARS-CoV-2 occurred in Wuhan, Hubei Province, China in December, 2019 (15–17). Initially, clinicians diagnosed this disease as virus-induced pneumonia based on blood tests and chest radiographs. Later, genomic and phylogenetic data analysis led to the recognition of the pathogen as a member of the *Coronaviridae* family (18). *Coronaviridae* family encompasses the largest known enveloped, single stranded RNA viruses with a genome size ranging from 25-32 kilo base pairs (Kb) (19,20). The family is divided into two subfamilies, the *Coronavirinae* and the *Toronavirinae*. The subfamily *Coronavirinae* is further organized genotypically and serologically into 4 genera: α, β, γ, and δ-CoVs (21). The *betacoronavirus* genus is comprised of the Severe Acute Respiratory Syndrome (SARS)-CoV which had been identified for the first time in 2002-2003 and the Middle East Respiratory Syndrome (MERS)-CoV in 2012. The genome sequences of SARS-Cov-2 has a 79.6% identity with SARS-CoV/ SARS-CoV-1 and 67.06%

identity with MERS-CoV, indicating that they belong to the *betacoronavirus* genus (22). All human coronaviruses are considered to be of zoonotic origin, with Chinese bats being the most likely host for SARS-CoV-2 (23–25). Genetically, about 96% identity was observed between SARS-CoV-2 and bat coronavirus (BatCoV RaTG13)(18). However, since bat habitats remain distanced from human life, an intermediate animal such as pangolin might have acted as an intermediate shuttle before transmitting to its human hosts (26–30).

The Chinese Center for Disease Control and Prevention (CDC) primarily suggested the Huanan local seafood market as the origin of the COVID-19 outbreak (31). Despite this claim, none of the animals in the area were tested positive for the virus. This indicated that the virus had already moved out of Wuhan, long before the outbreak came under spotlight. Since then the control of viral transmission through non-therapeutic interventions suggested by the World Health Organization (WHO) had been attempted (32). However, the violation of these preventive measures and absence of proper antiviral therapeutics and vaccinations led to an uncontrollable global transmission of the disease. The virus proliferated rapidly both inside and outside of China and finally reached each and every county of the world. In March 2020, the disease was declared as a global pandemic by the World Health Organization (WHO) (15). Although, at the beginning of the pandemic, the intensity of the disease was higher in the Europe and the America but later it also spread to Asian and South-East Asian countries (33–35).

Previously, the world went through three waves of the deadly Spanish flu until it subsided in 1919 while the second wave being the deadliest. The reason behind this fatal phenomenon was the rapid dispersion of the virus to every corner of the world (36) . A similar pattern can be observed in the case of COVID-19. By late 2020s and early 2021, a resurgence of infections was experienced by most countries including the United States, Brazil, Belgium, France, UK, Germany, as well as

most of the Asian countries (37–39). Remarkably India, which survived the first wave relatively unscathed, is currently suffering from a spine-chilling situation with a higher mortality rate than most other countries seeing more than 2000 deaths per day (40).

A well-established fact is that all viruses undergo genetic drift over time due to selection pressure and give rise to a number of variants that challenge any pandemic response (41,42) Therefore, understanding the current variants are crucial in restricting the mode of transmission and developing new therapeutics against them. Multiple variants have been identified around the world so far including B.1.1.7 (43), B.1.351 (44), P.1 (45), B.1.427/B.1.429 (46) and B.1.617(47). The B.1.1.7 variant was first detected in the United Kingdom around September, 2020. Three different types of mutations were observed in this variant which were present in the receptor binding domain of the spike protein, the 69/70 deletion and the P681H mutation near the S1/S2 furin cleavage sites. The alpha or kent variant turns out to be mutating again. In December 2020, B.1.351 was spotted as the predominant variant in South Africa (48). The variant, sharing some mutations with B.1.1.7 also had multiple mutations in their spike proteins such as K417N and E484K (49). The P.1 variant was first identified in Japan in a few travelers coming from Brazil in early January, 2021 (50). B.1.427 and B.1.429 variants were first detected in California in February 2021 (46). B.1.617.2 is the daunting variant of coronavirus that originated in India and has been circulating globally in at least 62 countries including the United States and United Kingdom (51). About 70% of the genome sequences submitted from India to GISAID constitute this variant. The major mutations in the delta variant includes substitution in the amino acid sequences of the spike protein (52,53).

Bangladesh, being one of the most densely populated countries of the world with over 160 million people and sharing a porous border with India, remains one of the most vulnerable countries for the second wave of the COVID-19 pandemic. The country with limited resources and scarce

healthcare facilities experiences major challenges while combating this transmission. The first case of this virus in the country was confirmed in two men coming from Italy and a female relative by the Institute of Epidemiology, Disease Control and Research (IEDCR) on March 7th, 2020 (54) Although many Bangladeshi citizens came from Wuhan beforehand, they were reported to be negative for SARS-CoV-2. As a response, the Bangladesh government took a number of preventive measures including nationwide lockdowns, imposing restrictions on international flights, strengthening of screening procedures, and shutting down of educational institutions and so on (55). Despite several rounds of lockdowns, the rate of infections continued to reach high levels. Correspondingly, it became the second most affected country in Southeast Asia. Near the end of the first wave, it began to drop gradually since November 2020. Although the rate declined to its lowest during January and February, 2021, the cases began to rise again (56).

The first complete genome sequencing of the SARS-CoV-2 in Bangladesh was announced by the Child Health Research Foundation on 12th May, 2020 (57). Soon after, the National Institute of Biotechnology announced the sequencing of SARS-CoV-2 genome by Sanger sequencing method (58). The SARS-CoV-2 genome sequencing effort in Bangladesh flourished afterwards and as a result, 1569 genomes have been sequenced by June 6, 2020.

The goal of this study was to probe all these sequences and find some crucial answers regarding the genomic evolution of the virus, predominant variants, difference between the first and the second wave and so on which would make it easier to comprehend the trajectory of the pandemic and suggest appropriate counter measures.

2. Materials and Methods

2.1 Retrieval of the SARS-CoV-2 Genome Sequences

Genomes of SARS-CoV-2 isolates were retrieved from the Global Initiative on Sharing All Influenza Data (GISAID) database (www.gisaid.org) (59). Isolates collected since the beginning of the COVID-19 pandemic till 31 Jan 2021 were considered as wave-1. (**Supplementary file 1**). and those collected between Feb 1, 2021 and Jun 6, 2021 were considered as wave-2 samples (**Supplementary file 2**).

2.2 Wave-1 and Wave-2 Mutation Analysis

The Wuhan genome reference sequence (NC_045512.2) was retrieved from NCBI GenBank (60). A GFF3 annotation file of the reference sequence (**Supplementary file 3**), generated by Giorgi was used for extracting the genomic coordinates of SARS-CoV-2 proteins (61). The sequences from wave-1 and wave-2 were aligned separately against the reference sequence using the NUCMER (version 4.0.0rc1) command line tool (62). A SARS-CoV-2 annotation algorithm, developed by Mecatelli and Giorgi (61), was employed to convert the outputs of alignments into lists of mutational events. Frequency and the rate of mutation per sample was calculated. All the SARS-CoV-2 sequences from both waves were classified based on the type of mutation. Specific coordinates of the mutations on the SARS-CoV-2 genome were also identified. Finally, alterations in the proteome of SARS-CoV-2 as a result of genomic variation were investigated. **Supplementary file 4** contains a detailed report on this mutation analysis for both COVID-19 pandemic waves in Bangladesh.

2.3 Clade and Variant Analysis for Wave-1 and Wave-2

For this analysis, both complete and incomplete sequences for wave-1 and wave-2 in the Bangladesh region were obtained from GISAID. The number of sequences for different clades was counted directly from this database. Assignment of different lineages for each sample from both waves was performed by pangolin (version v3.0.5, lineages version 2021-06-05) web server (https://pangolin.cog-uk.io/). For all sequences, Greek Alphabet names of relevant lineages, as well as their classes (VOC for variants of concern, VOI for variants of interest and Unclassified for other variants), were also ascribed. Percentage of occurrences for different clades and top ten variants were calculated via R commands. Finally, comparative plots were generated by using the ggplot2 package in R (63) to describe the distribution of SARS-CoV-2 clades and variants in Bangladesh.

2.4 Molecular Docking between Spike Protein and ACE2

The reference sequence of the Receptor Binding Domain (RBD) of SARS-CoV-2 spike glycoprotein (S) was taken from UniProt (https://www.uniprot.org/) (UniProt ID: P0DTC2) and was manually mutated to generate the sequence of the variants B.1.1.7, B.1.351, and B.1.617 which were most common in Bangladesh. 3D models of all the sequences were built using Robetta (64) . The structure of the human ACE2 receptor was extracted from the RCSB PDB (PDB ID: 6M0J). Docking between S-RBD and ACE2 was conducted by GalaxyTongDock_A server (65). Following protein-protein docking, the generated models with the highest docking scores and cluster size were selected and submitted to PROtein binDIng enerGY prediction (PRODIGY) to calculate the binding affinity of the protein-protein complexes at physiological temperature (37 °C) (66).

2.5 Molecular Dynamic Simulation of ACE2-Spike Protein Complex

In order to evaluate the evaluate the stability of the complex between the ACE2 receptor and the SARS-CoV-2 Spike protein (Reference and the variants B.1.1.7, B.1.351, and B.1.617) under physiological conditions, 50 ns molecular dynamics simulation was executed with GROningen MAchine for Chemical Simulations aka GROMACS (version 5.1.1) (67). The GROMOS96 43a1 force-field was used for the simulation (68). 300 K temperature, pH 7.4, and 0.9% NaCl solution was used to define the physiological condition of the system. A dodecahedral box with its edges at 1 nm distance from the protein surface was drawn and the system was solvated with SPC (simple point charge) water model . Using the genion module inherent to GROMACS, the overall charge of the system was neutralized by adding 23 NA ions. The steepest descent minimization algorithm was utilized to carry out energy minimization of the system. Isothermal-isochoric (NVT) equilibration of the system was carried out for 100 ps with short-range electrostatic cutoff value of 1.2 nm. Then the Isobaric (NPT) equilibration of the system was carried out for 100 ps as well with short-range van der Waals cutoff fixed at 1.2 nm. Finally a 50 ns molecular dynamic simulation was done using periodic boundary conditions and time integration step of 2 fs. After every 100 ps, the energy of the system was recorded. The Particle Mesh Ewald (PME) method was employed for calculating the long range electrostatic potential. The short-range van der Waals cutoff was set to 1.2 nm. The simulation temperature was maintained using modified Berendsen thermostat while the pressure was made constant using the Parrinello-Rahman algorithm. An interval of 100 ps was used each snapshot for analyzing the trajectory data. Eventually the trajectory information gathered throughout the simulation were concatenated to calculate and plot root mean square deviation (RMSD), root mean square fluctuation (RMSF), radius of gyration (Rg) and solvent accessible surface area (SASA) data. MD simulations were performed on the

"bioinfo-server" running on Ubuntu 18.4.5 operating system located at the Bioinformatics Division, National Institute of Biotechnology.

In order to evaluate structural stability, Root Mean Square Deviation (RMSD) calculation was performed. The "rms" module built into the GROMACS software was utilized to extract RMSD information throughout the course of the simulation. The result can be plotted graphically using the Xmgrace package.

Room Mean Square Fluctuation (RMSF) measurement was used to determine the flexibility of local structures within the ACE2-Spike protein complex. The higher RMSF values corresponded to higher flexibility of a region. RMSF calculations were carried out using the "rmsf" module and the figures were generated using Xmgrace.

To determine the degree of compactness, the radius of gyration of the complex was calculated. A relatively steady value of radius of gyration means stable folding of a protein. Fluctuation of radius of gyration implies the unfolding of the protein. The "gyrate" module was used to generate the radius of gyration graphs for our proteins.

Hydrophobic interactions composed of non-polar amino acids are crucial for maintaining the stability of the hydrophobic core of proteins. They do so by covering the non-polar amino acids within the hydrophobic cores and keeping them at a distance from the solvent. Solvent Accessible Surface Area (SASA) is used in molecular dynamic simulations to predict the hydrophobic core stability of proteins. In this study, SASA was calculated using the "sasa" module and the resulting graph was visualized using Xmgrace.

3. Results

3.1 SARS-CoV-2 Genomes from Bangladesh

From the first instance of SARS-CoV-2 genome submission from Bangladesh (May 12, 2020) to the time of the present study (June 6, 2021), the GISAID database recorded 1569 SARS-CoV-2 isolates from Bangladesh. According to our analysis, a total of 1074 samples belonged to wave-1 and 495 samples to wave-2.

3.2 Frequency of SARS-CoV-2 Mutations in Bangladesh

In comparison to the Wuhan reference sequence, all sequences from both waves appeared to have two or more mutations (**Supplementary file 5**). The average number of mutations per sample was found to differ significantly between the two waves (**Fig 1**) based on two sided t-test $p = 2.2 \times 10^{-16}$. The average rate of mutation in wave-2 samples (30.79%) was substantially higher than the wave-1 samples (12.32%). In the case of wave-1 (1074 samples), most of the sequences possessed 6 to 17 mutations per sample, while the majority of the sequences in wave-2 (495 samples) tended to have 28 to 38 mutations (**Fig 2**).

Fig 1: Density plot of average mutations per sample in case of wave-1 and wave-2. The Red line and Blue line represent the average value of mutation per sample for wave-1 and wave-2 respectively. Wave-2 samples generally possessed a higher number of mutations per sample than wave-1.

Fig 2: Number of mutations per sample for wave-1 and wave-2. The majority of sequences from wave-1 had 6 to 17 mutations per sample. On the other hand, most of the samples of wave-2 possess on average 28 to 38 mutations.

3.3 Type of SARS-CoV-2 Mutations in Bangladesh

The occurrence of several classes of mutations, as well as the percentages of each class for both waves, are documented in the **Supplementary file 6**. Single-nucleotide polymorphisms (SNPs) seemed to be highly prevalent in both cases (58.89% in wave-1 and 61.5% in wave-2) (**Fig 3**). Extragenic mutations were also found to some extent, but all were either in 5'-UTR or in 3'-UTR regions. Wave-2 cases (943 events, 6.19%) had considerably more deletion events than wave-1 instances (115 events, 0.87%). During the first surge of the pandemic, the insertion (0.03%), deletion_stop(0.01%), or insertion_stop(0.01%) events took place in a small fraction of cases, but not at all in the second phase.

Fig 3: Distribution of mutation classes between two waves of COVID-19 in Bangladesh. SNPs, silent SNPs, and extragenic mutations were similarly abundant in both waves. But, sequences of wave-2 had a significant amount of deletion events.

All mutational events were also classified into different variant types to explain the higher frequency of SNPs (**Supplementary file 7**). Though both SNP transitions (purine > purine, pyrimidine > pyrimidine) and transversion (purine > pyrimidine and vice versa) were observed among all samples, C>T transition was the most frequent mutation in both waves (**Fig 4**). The percentage of occurrence of this transitional event was 45.75% in wave-1 and 42.44% in wave-2. While A>G transition is the second most common event in wave-1(12.53%), G>T transversion possessed this place in the case of wave-2(13.57%). Oligonucleotide deletion was also commonly present in the samples from wave-2. In the second wave of the COVID-19 pandemic in Bangladesh, two oligonucleotide deletion events (TCTGGTTTT and CTTGCTTTA) appeared to be much more pervasive (2.46% and 1.63% respectively).

Fig 4: Most frequent mutational events per type between the two waves. The C>T transition was highly prevalent in both waves. While A>G transition is the second most abundant event in

wave-1, samples from wave-2 had G>T transversion event.

3.4 Genomic Location of the SARS-CoV-2 Mutations

The presence of mutational changes in specific coordinates of the SARS-CoV-2 genome sequences was also analyzed in this study (**Supplementary file 8**). In both waves, the A23403G, C3037T, C14408T, and C241T mutations showed a similar pattern of abundance (**Fig 5)**. Although the GGG28881AAC trinucleotide substitution was the 5th most prevailing event in the case of Wave-1, its existence was much lower in the case of Wave-2 (only 0.75%). Rather TCTGGTTTT11288 deletion was substantially more common in the second phase, which is consistent with previous findings of this study.

Fig 5: Most frequent mutations at the nucleotide level in wave-1 and wave-2. The first four nucleotide events were the most widespread for both waves in Bangladesh. Among them, A23403G, C3037T, and C241T mutations are characteristic features of G clade and its derivatives. Wave-2 showed a substantial frequency of the TCTGGTTTT oligonucleotide deletion at 11288 position.

3.5 Impact of Mutations on the SARS-CoV-2 Proteome

We also summarized the impacts of these mutations on the protein sequence of SARS-CoV-2 in the final step of this mutational investigation (**Supplementary file 9**). The D614G (aspartate to glycine in the 614th amino acid) mutation in the spike protein of SARS-CoV-2 is caused by the most predominant nucleotide transversion (A > G) in the 23,403rd position. This mutation, a characteristic feature of the G-clade of SARS-CoV-2 genome, was observed in the highest frequency in the samples of both waves (**Fig 6**). From this observation it can be said that the G-

clade of this virus was ubiquitous in Bangladesh in the case of both waves, which we have also explicated later through the clade distribution analysis. Despite sorting by frequency, the pattern of dominance of S:D614G, NSP3:F106F, NSP12b:P314L and, 5'UTR:241 mutations was identical in both waves, however in wave-1, these mutations were at a somewhat greater percentage (8.01%, 7.82%, 7.70% and, 7.48% respectively) than other modifications. On the other hand, these mutations accounted for 3.24%, 3.22%, 3.11%, and 2.93% of total amino acid alteration events from wave-2 samples.

Fig 6: Number of most frequent protein level alterations among two waves in Bangladesh. The four most frequent amino acid substituting events had the same type of distribution for both waves. Besides, some mutations in spike and nucleocapsid protein (e.g., S:N501Y, N:T205I, S:D80A) were found in a significant amount in wave-2. Furthermore, the ORF3a:Q57H variant, which is a marker variant for GH clade, was also very common in this case.

3.6 Distribution of SARS-CoV-2 Clades in Bangladesh

Furthermore, the distribution of various SARS-CoV-2 clades and most frequent variants was also compared across two waves in Bangladesh (**Supplementary file 10**). Throughout the pandemic in Bangladesh, the G-clade and its derivatives (GH, GV, GR, GRY) continued to be dominant (**Fig 7**). Although the GR clade was predominant during wave-1(75.86%), in wave-2 the GH clade took the lead (61.26%). However, the percentage of other G-clades was pretty much similar in both phases of the pandemic. On the contrary, in wave-1, the L, O, and S clades had a very low frequency, and in wave-2, the L and S clades disappeared. The variants from the B lineage were extremely common in wave-1, with B.1.1.25 accounting for 72.46% of the total (**Fig 8**). Besides, the alpha variant (B.1.1.7), a variant of concern, also showed up to some extent. In the scenario of

wave-2, however, the B.1.351.3 (57.44%) variant dominated throughout the entire time frame. During this wave, there was also a progressive increase of VOC variants (alpha, beta, delta).

Fig 7: Comparative distribution of different clades in two waves of COVID-19 pandemic in Bangladesh. G clade and its descendants dominated throughout the pandemic. But GR clade in the case of wave-1 and GH clade in the case of wave-2 were the most prevalent ones. In the second wave, the L, O, and S clades were almost completely lost.

Fig 8: Prevalence of different variants (in percentage) observed in wave-1 and 2 of COVID-19 pandemic in Bangladesh. Almost all SARS-CoV-2 variants of wave-1 were from B lineage. On the other hand, wave-2 had an increased number of VOC variants. The most prevalent B.1.351.3 variant of this wave is a derivative of the beta (B.1.351) variant.

3.7 Molecular Docking between the ACE2 and Spike Protein

Molecular docking between human ACE2 receptor and the receptor binding domain (RBD) of Spike protein found in the reference and the 3 most commonly found variants namely, B.1.1.7, B.1.351, B.1.617 revealed that the variants B.1.351 and B.1.617 had the two highest binding affinities respectively. Notably, the variant B.1.617 exhibited the highest docking score (**Table 3**).

Table 3: Results of Molecular Docking between SARS-CoV 2 Spike glycoprotein Receptor Binding Domain (RBD) and human ACE2 receptor. The binding affinity was measured in physiological temperature (37° C).

Spike protein variant ACE2	TongDock_A docking score	TongDock_A Cluster size	Binding affinity, ΔG (kcal mol-1)	Kd (M) at 37.0 ℃

Reference	1262.964	34	-13.4	3.5E-10
B 1.1.7	1293.348	36	-12.8	1.0E-09
B.1.351	1447.344	32	-16.4	2.9E-12
B.1.617	1541.675	39	-16.2	3.5E-12

3.8 Molecular Dynamic Simulation of the ACE2-Spike Protein Complex

Protein backbone RMSD analysis of reference spike protein and the variants exhibited marked differences. The reference protein periodically showed large deviations until it attained stability at around 42 ns. The variant B.1.1.7 was much more stable. Despite some initial fluctuations it assumed stable conformation gradually after 20 ns. B.1.351 on the other hand was very stable since the beginning. However there were a number of spikes in between 34 and 40 ns after which the complex stabilized again. Among the four complexes tested, the one involving the variant B.1.617 was the most stable of all. It remained highly stable throughout the simulation except there was a rise in RMSD at 36-46 ns period. However it maintained a steady value within this period as well (**Fig 9a**).

Fig 9a: RMSD analysis of (I) Reference-ACE2 (II) B.1.1.7-ACE2 and (III) B.1.351-ACE2 complex (IIV) B.1.617-ACE2 complex. The last one appeared to be most stable among all

Upon evaluation of the detailed residual atomic fluctuations through RMSF calculation of the protein Cα atoms, it was apparent that both the reference and the variants B.1.351, and B.1.617 were very similar in the sense that the atoms near the end of the complex were more flexible than the rest of the complex (**Fig 9b**).

Fig 9b: RMSF values of (I) Reference-ACE2 (II) B.1.1.7-ACE2 and (III) B.1.351-ACE2 complex (IIV) B.1.617-ACE2 complex. Except B.1.1.7, the rest of the variants showed very regional flexibility.

The Rg graphs revealed a very similar pattern found in the RMSD graphs. The variant B.1.617 remained in compact state throughout the simulation with a period of unfolding at 36-46 ns. The reference protein unfolded at regular intervals and often to a high degree. The variant B.1.1.7 folded steadily while B.1.351 unfolded abruptly several times from 34-40 ns and remained otherwise rest of the time (**Fig 9c**).

Fig 9c: Rg measurement of (I) Reference-ACE2 (II) B.1.1.7-ACE2 and (III) B.1.351-ACE2 complex (IIV) B.1.617-ACE2 complex. The B.1.617-ACE2 complex remained more compact than the rest.

SASA values provided a measure of the complex's susceptibility to disruption of their hydrophobic core by water . For all four complexes, the SASA declined gradually over time. However, the greatest reduction took place in the case of the variant B.1.617 (**Fig 9d**).

Fig 9d: Solvent Accessibility Area calculation of (I) Reference-ACE2 (II) B.1.1.7-ACE2 and (III) B.1.351-ACE2 complex (IIV) B.1.617-ACE2 complex. Although solvent accessibility area gradually declined gradually for all 4 complex, the B.1.617-ACE2 complex lost solvent accessibility area the most.

4. Discussion

The first case of COVID-19 in Bangladesh was identified on migrants returning from Italy at the beginning of March (69). Since then, according to the official record (as of Jun 15, 2021) about

829K people have been affected and over 13K died of this virus in Bangladesh. Meanwhile, the virus has affected over 177 million individuals globally, with over 3.8 million deaths (1). Since the first patient had been identified, COVID-19 cases were found regularly in Bangladesh throughout the year 2020. The number of COVID-19 cases in neighboring India has been rising rapidly since March 2021. Inevitably, the number of SARS-CoV-2 cases in Bangladesh is also on the upswing. In this study, a comparative genomic analysis was performed to track the dynamics of SARS-CoV-2 evolution between the two waves of the COVID-19 pandemic in Bangladesh.

The rate of mutation in SARS-CoV-2 (~2 nucleotides/month) is far lower than that of influenza (4 nucleotides/month) or HIV (8 nucleotides/month), yet its distinct genomic regions and proteins are mutating at significantly variable rates (70,71). The frequency of these mutations alters considerably depending on the geographical location with time as well. In this study, a significant rise in the rate of mutation was observed in wave-2 samples of Bangladesh compared to the wave-1. A similar pattern was observed in the instance of SARS-CoV-2 pandemic waves in Hiroshima, Japan (72). Although both waves in Bangladesh had a higher incidence of amino acid altering SNPs, wave-2 tended to have a higher number of deletion events (**Fig 3**). Such recurring recurrent deletion events in the SARS-CoV-2 genome had been reported to facilitate its transmission with altered antigenicity and antibody escape mechanism (73). Furthermore, despite the fact that C>T transitions prevailed in both waves, G>T transversion was rather frequent in wave-2 (**Fig 4**). This transversion provoked the G25563T nucleotide mutation event (ORF3a:Q57H in protein level) in the SARS-CoV-2 genome, which was a marker variant for GH clade (74). This might explain why the GH clade was observed to be more apparent in Bangladesh during Wave 2. Different marker variants for GISAID clades and lineages have been listed in **Table 1**. The phylogenetic clusters derived from the statistical distribution of SARS-CoV-2 genomic distances have been used to

define these clade classifications in GISAID (75).

Table 1: List of the Marker Variants for GISAID Clade and Lineage (76).

GISAID Clade	Lineage	Nucleotide Events	Protein Events
S	A	C8782T, T28144C	NS8:L84S
L	B	Reference Genome from Wuhan	
V	B.2	G11083T, G26144T	NSP6:L37F, NS3:G251V
G	B.1	C241T, C3037T, A23403G	S:D614G
GH	B.1.*	C241T, C3037T, A23403G, G25563T	S:D614G, ORF3a3:Q57H [A17]
GR	B.1.1	C241T, C3037T, A23403G, G28882A	S:D614G, N:G204R
GV	B.1.177	C241T, C3037T, A23403G, C22227T	S:D614G, S:A222V
GRY	B.1.7	C241T, C3037T, 21765-21770del, 21991-21993del, A23063T, A23403G, G28882A	S:H69del, S:V70del, S:Y144del, S:N501Y, S:D614G,

			N:G204R

Fig 3: Distribution of mutation classes between two waves of COVID-19 in Bangladesh. SNPs, silent SNPs, and extragenic mutations were similarly abundant in both waves. But, sequences of wave-2 had a significant amount of deletion events.

Fig 4: Most frequent mutational events per type between the two waves. The C>T transition was highly prevalent in both waves. While A>G transition is the second most abundant event in wave-1, samples from wave-2 had G>T transversion event.

The second and third most common SNPs in Bangladesh were silent and extragenic SNPs, respectively. Even though these SNPs do not alter the protein sequence directly, they have a major impact on the efficiency of translation and transcription. SNPs in the 5'-UTR, in particular, can influence the virus's transcription and replication processes by altering the folding of genomic RNA (75). The A23403G, G3037T, C241T nucleotide variants as well as S: D614G, ORF3a:Q57H, N:G204R protein variants were equally abundant in both waves (**Fig 6**). All of these are marker variants for the G clade and its derivatives (**Table 1**), which explains why Bangladesh experienced a greater distribution of these clades (**Fig 7**).

Fig 6: Number of most frequent protein level alterations among two waves in Bangladesh. The four most frequent amino acid substituting events had the same type of distribution for both waves. Besides, some mutations in spike and nucleocapsid protein (e.g., S:N501Y, N:T205I, S:D80A) were found in a significant amount in wave-2. Furthermore, the ORF3a:Q57H variant, which is a marker variant for GH clade, was also very common in this case.

Fig 7: Comparative distribution of different clades in two waves of COVID-19 pandemic in Bangladesh. G clade and its descendants dominated throughout the pandemic. But GR clade in the case of wave-1 and GH clade in the case of wave-2 were the most prevalent ones. In the second wave, the L, O, and S clades were almost completely lost.

In May 2021, the World Health Organization (WHO) recommended adopting Greek Alphabet letters to name several important SARS-CoV-2 variants (**Table 2**). The most widely available B.1.351.3 variant in wave-2 in Bangladesh is a sublineage of beta, a VOC variant first detected in South African samples. On the other hand, the delta variant, which is driving a catastrophic pandemic in neighboring country India, is the third most frequent variant in this wave in Bangladesh (**Fig 8**).

Table 2: Naming SARS-CoV-2 variants by World Health Organization (WHO) (77)

**WHO Label**	**Lineage**	**Variant Class**	**First Detected Samples**
Alpha	B.1.1.7	VOC	UK, Sep-2020
Beta	B.1.351	VOC	South Africa, May-2020
Gamma	P.1	VOC	Brazil, Nov-2020
Delta	B.1.617.2	VOC	India, Oct-2020
Epsilon	B.1.427, B.1.429	VOI	USA, Mar-2020
Zeta	P.2	VOI	Brazil, Apr-2020
Eta	B.1.525	VOI	Several Countries, Dec-2020

Theta	P.3	VOI	Philippines, Jan-2021
Iota	B.1.526	VOI	USA, Nov-2020
Kappa	B.1.617.1	VOI	India, Oct-2020

Fig 8: Prevalence of different variants (in percentage) observed in wave-1 and 2 of COVID-19 pandemic in Bangladesh. Almost all SARS-CoV-2 variants of wave-1 were from B lineage. On the other hand, wave-2 had an increased number of VOC variants. The most prevalent B.1.351.3 variant of this wave is a derivative of the beta (B.1.351) variant.

The importance of the interaction between the ACE2 receptor and the SARS-CoV-2 spike protein is paramount in understanding the pathogenesis of COVID-19 infection (78). Molecular docking and dynamics simulations are one of the key tools for getting in-depth insights into these interactions (79–81).

Molecular docking experiment showed that the Spike protein of the variants B.1.351 and B.1.617 bound most tightly to the ACE2 receptor. From the 50 ns molecular dynamics simulation carried out in GROMACS, the complex between the ACE2 receptor and the Spike protein variant of B.1.617 was found to be most stable considering its structural deviation, local area flexibility, state of folding, and susceptibility to disruption by solvents (**Fig 9**). These findings coincide with those reported in other studies and the enhanced Spike protein stability of the B.1.617 is likely to contribute to the efficiency of transmission of SARS-CoV-2 (82–84).

Fig 9a: RMSD analysis of (I) Reference-ACE2 (II) B.1.1.7-ACE2 and (III) B.1.351-ACE2 complex (IIV) B.1.617-ACE2 complex. The last one appeared to be most stable among all.

Fig 9b: RMSF values of (I) Reference-ACE2 (II) B.1.1.7-ACE2 and (III) B.1.351-ACE2 complex (IIV) B.1.617-ACE2 complex. Except B.1.1.7, the rest of the variants showed very regional flexibility.

Fig 9c: Rg measurement of (I) Reference-ACE2 (II) B.1.1.7-ACE2 and (III) B.1.351-ACE2 complex (IIV) B.1.617-ACE2 complex. The B.1.617-ACE2 complex remained more compact than the rest.

Fig 9d: Solvent Accessibility Area calculation of (I) Reference-ACE2 (II) B.1.1.7-ACE2 and (III) B.1.351-ACE2 complex (IIV) B.1.617-ACE2 complex. Although solvent accessibility area gradually declined gradually for all 4 complex, the B.1.617-ACE2 complex lost solvent accessibility area the most.

5. Conclusions

The current study revealed significant genomic and proteomic differences in the SARS-CoV-2 viral isolates circulating within the perimeters of Bangladesh between the first and the second wave of the COVID-19 pandemic. They differ in terms of distribution of clades, mutations, variants, rate of mutations, and even in terms of their interactions with the host ACE2 receptor. The study found evidence that the B.1.617 lineage of the virus is likely to be more infectious than others. Notably, any existence of a domestic variant is yet to be detected. Therefore, if Bangladesh can shield itself from the arrival of SARS-CoV-2 variants from outside for a substantial period, the COVID-19 pandemic in the country is likely to come to an end.

Acknowledgements

This research work did not receive any funding.

References

1. Coronavirus Graphs: Worldwide Cases and Deaths - Worldometer [Internet]. [cited 2021 Jun 15]. Available from: https://www.worldometers.info/coronavirus/worldwide-graphs/
2. SL K, A M, A M, SM L. The Threat of Pandemic Influenza. The Threat of Pandemic Influenza. National Academies Press; 2005.
3. Muscatello DJ, McIntyre PB. Comparing mortalities of the first wave of coronavirus disease 2019 (COVID-19) and of the 1918–19 winter pandemic influenza wave in the USA. Int J Epidemiol. 2020 Sep;
4. Hasanul Banna Siam M, Mahbub Hasan M, Raheem E, Hasinur Rahaman Khan M, Siddiqee MH, Sorowar Hossain M. Insights into the first wave of the COVID-19 pandemic in Bangladesh: Lessons learned from a high-risk country. medRxiv. 1101;2020.08.05.20168674.
5. Salzberger B, Buder F, Lampl B, Ehrenstein B, Hitzenbichler F, Holzmann T, et al. Epidemiology of SARS-CoV-2. Infection [Internet]. 2020 Oct 8 [cited 2020 Dec 4]; Available from: https://pubmed.ncbi.nlm.nih.gov/33034020/
6. Moore G, Rickard H, Stevenson D, Bou PA, Pitman J, Crook A, et al. Detection of SARS-CoV-2 within the healthcare environment: A multicentre study conducted during the first wave of the COVID-19 outbreak in England. J Hosp Infect. 2020 Sep;2020.09.24.20191411.
7. 24. Ryu S, Ali ST, Noh E, Kim D, Lau EHY, Cowling BJ. Transmission dynamics and control of two epidemic waves of SARS-CoV-2 in South Korea. BMC Infectious Diseases.

2021 May 26;21(1).

8. Grubaugh ND, Ladner JT, Kraemer MUG, Dudas G, Tan AL, Gangavarapu K, et al. Genomic epidemiology reveals multiple introductions of Zika virus into the United States. Nature. 2017 Jun;546(7658):401–5.

9. Ladner JT, Grubaugh ND, Pybus OG, Andersen KG. Precision epidemiology for infectious disease control. Vol. 25, Nature Medicine. Nature Publishing Group; 2019. p. 206–11.

10. Kalinich CC, Jensen CG, Neugebauer P, Petrone ME, Peña-Hernández M, Ott IM, et al. Real-time public health communication of local SARS-CoV-2 genomic epidemiology. PLOS Biology. 2020 Aug 21;18(8):e3000869.

11. Deng X, Gu W, Federman S, du Plessis L, Pybus OG, Faria NR, et al. Genomic surveillance reveals multiple introductions of SARS-CoV-2 into Northern California.

12. Meredith LW, Hamilton WL, Warne B, Houldcroft CJ, Hosmillo M, Jahun AS, et al. Rapid implementation of SARS-CoV-2 sequencing to investigate cases of health-care associated COVID-19: a prospective genomic surveillance study. Lancet Infect Dis. 2020 Nov 1;20(11):1263–72.

13. An integrated national scale SARS-CoV-2 genomic surveillance network. The Lancet Microbe. 2020 Jul;1(3):e99–100.

14. Resende PC, Delatorre E, Gräf T, Mir D, Motta F do C, Appolinario LR, et al. Genomic surveillance of SARS-CoV-2 reveals community transmission of a major lineage during the early pandemic phase in Brazil. bioRxiv. 2020 Jun;2020.06.17.158006.

15. Morens DM, Breman JG, Calisher CH, Doherty PC, Hahn BH, Keusch GT, et al. The Origin

of COVID-19 and Why It Matters. The American Journal of Tropical Medicine and Hygiene. 2020 Jul 22;103(3).

16. Bolsen T, Palm R, Kingsland JT. Framing the Origins of COVID-19. Science Communication [Internet]. 2020 Sep 10; Available from: https://www.ncbi.nlm.nih.gov/pmc/articles/PMC7484600/

17. Burki T. The origin of SARS-CoV-2. The Lancet Infectious Diseases. 2020 Sep;20(9):1018–9.

18. Zhou P, Yang X Lou, Wang XG, Hu B, Zhang L, Zhang W, et al. A pneumonia outbreak associated with a new coronavirus of probable bat origin. Nature. 2020 Mar;579(7798):270–3.

19. Khailany RA, Safdar M, Ozaslan M. Genomic characterization of a novel SARS-CoV-2. Gene Reports. 2020 Jun 1;19:100682.

20. Naqvi AAT, Fatima K, Mohammad T, Fatima U, Singh IK, Singh A, et al. Insights into SARS-CoV-2 genome, structure, evolution, pathogenesis and therapies: Structural genomics approach. Vol. 1866, Biochimica et Biophysica Acta - Molecular Basis of Disease. Elsevier B.V.; 2020. p. 165878.

21. Payne S. Family Coronaviridae. In: Viruses. Elsevier; 2017. p. 149–58.

22. Hossain MU, Bhattacharjee A, Emon MTH, Chowdhury ZM, Mosaib MG, Mourin M, et al. Recognition of plausible therapeutic agents to combat COVID-19: An omics data based combined approach. Gene. 2021 Mar;771.

23. Chang C, Sue S-C, Yu T, Hsieh C-M, Tsai C-K, Chiang Y-C, et al. Modular organization

of SARS coronavirus nucleocapsid protein. Journal of Biomedical Science [Internet]. 2005 Oct 14 [cited 2020 Sep 30];13(1):59–72. Available from: https://www.ncbi.nlm.nih.gov/pmc/articles/PMC7089556/

24. Chen N, Zhou M, Dong X, Qu J, Gong F, Han Y, et al. Epidemiological and clinical characteristics of 99 cases of 2019 novel coronavirus pneumonia in Wuhan, China: a descriptive study. Lancet. 2020 Feb 15;395(10223):507–13.

25. Woo PCY, Lau SKP, Lam CSF, Lau CCY, Tsang AKL, Lau JHN, et al. Discovery of Seven Novel Mammalian and Avian Coronaviruses in the Genus Deltacoronavirus Supports Bat Coronaviruses as the Gene Source of Alphacoronavirus and Betacoronavirus and Avian Coronaviruses as the Gene Source of Gammacoronavirus and Deltacoronavirus. J Virol. 2012 Apr 1;86(7):3995–4008.

26. Lam TTY, Jia N, Zhang YW, Shum MHH, Jiang JF, Zhu HC, et al. Identifying SARS-CoV-2-related coronaviruses in Malayan pangolins. Nature. 2020;583(7815).

27. Hong B, Lai X, Chen Y, Luo T, An X, Song L, et al. SARS-CoV-2 and Malayan pangolin coronavirus infect human endoderm, ectoderm and induced lung progenitor cells. bioRxiv [Internet]. 2020 Sep 25 [cited 2020 Nov 19];2020.09.25.313270. Available from: https://doi.org/10.1101/2020.09.25.313270

28. Zhang T, Wu Q, Zhang Z. Probable Pangolin Origin of SARS-CoV-2 Associated with the COVID-19 Outbreak. Curr Biol. 2020 Apr 6;30(7):1346-1351.e2.

29. Han GZ. Pangolins Harbor SARS-CoV-2-Related Coronaviruses. Vol. 28, Trends in Microbiology. Elsevier Ltd; 2020. p. 515–7.

30. Xiao K, Zhai J, Feng Y, Zhou N, Zhang X, Zou J-J, et al. Isolation of SARS-CoV-2-related coronavirus from Malayan pangolins. Nature [Internet]. 2020 May 7 [cited 2020 May 10];1–7. Available from: https://www.nature.com/articles/s41586-020-2313-x

31. Adhikari SP, Meng S, Wu YJ, Mao YP, Ye RX, Wang QZ, et al. Epidemiology, causes, clinical manifestation and diagnosis, prevention and control of coronavirus disease (COVID-19) during the early outbreak period: A scoping review. Vol. 9, Infectious Diseases of Poverty. BioMed Central Ltd.; 2020. p. 1–12.

32. Böhmer MM, Buchholz U, Corman VM, Hoch M, Katz K, Marosevic D V., et al. Investigation of a COVID-19 outbreak in Germany resulting from a single travel-associated primary case: a case series. Lancet Infect Dis. 2020 Aug;20(8):920–8.

33. Grasso M, Klicperová-Baker M, Koos S, Kosyakova Y, Petrillo A, Vlase I. The impact of the coronavirus crisis on European societies. What have we learnt and where do we go from here? – Introduction to the COVID volume. European Societies. 2021 Jan 27;23(sup1):S2–32.

34. Salimi A, ElHawary H, Diab N, Smith L. The North American Layman's Understanding of COVID-19: Are We Doing Enough? Frontiers in Public Health. 2020 Jul 3;8.

35. Barai MK. COVID 19 in South Asia and the Way Forward: An Introduction. South Asian Survey. 2021 Mar;28(1):7–19.

36. Martini M, Gazzaniga V, Bragazzi NL, Barberis I. The Spanish Influenza Pandemic: A lesson from history 100 years after 1918. J Prev Med Hyg. 2019;60(1):E64–7.

37. James N, Menzies M, Radchenko P. COVID-19 second wave mortality in Europe and the

United States. Chaos: An Interdisciplinary Journal of Nonlinear Science. 2021 Mar;31(3):031105.

38. de Souza FSH, Hojo-Souza NS, da Silva CM, Guidoni DL. Second wave of COVID-19 in Brazil: younger at higher risk. European Journal of Epidemiology [Internet]. 2021 Apr 21 [cited 2021 Jun 17];1–3. Available from: https://www.ncbi.nlm.nih.gov/pmc/articles/PMC8058142/

39. Post LA, Lin JS, Moss CB, Murphy RL, Ison MG, Achenbach CJ, et al. SARS-CoV-2 Wave Two Surveillance in East Asia and the Pacific: Longitudinal Trend Analysis. Journal of Medical Internet Research. 2021 Feb 1;23(2):e25454.

40. Menon V, Kar SK, Ransing R, Arafat SMY. Impending Second Wave of COVID-19 Infections: What India Needs to Do? Asia Pacific Journal of Public Health. 2021 Mar 5;101053952199886.

41. Robinson J, Banerjee I, Leclézio A, Sathian B. COVID-19 and mutations a threat level assessment. Nepal J Epidemiol. 2021 Mar;11(1):983–7.

42. Zhou W, Wang W. Fast-spreading SARS-CoV-2 variants: challenges to and new design strategies of COVID-19 vaccines. Signal Transduct Target Ther. 2021 Dec;6(1):226.

43. Davies NG, Jarvis CI, van Zandvoort K, Clifford S, Sun FY, Funk S, et al. Increased mortality in community-tested cases of SARS-CoV-2 lineage B.1.1.7. Nature [Internet]. 2021 May 13 [cited 2021 Jun 15];593(7858):270–4. Available from: https://doi.org/10.1038/s41586-021-03426-1

44. Zhou D, Dejnirattisai W, Supasa P, Liu C, Mentzer AJ, Ginn HM, et al. Evidence of escape

of SARS-CoV-2 variant B.1.351 from natural and vaccine-induced sera. Cell. 2021 Feb;

45. Sabino EC, Buss LF, Carvalho MPS, Prete CA, Crispim MAE, Fraiji NA, et al. Resurgence of COVID-19 in Manaus, Brazil, despite high seroprevalence. The Lancet [Internet]. 2021 Jan 27 [cited 2021 Feb 3];0(0). Available from: https://www.thelancet.com/journals/lancet/article/PIIS0140-6736(21)00183-5/fulltext

46. McCallum M, Bassi J, Marco A De, Chen A, Walls AC, Iulio J Di, et al. SARS-CoV-2 immune evasion by variant B.1.427/B.1.429. bioRxiv Prepr Serv Biol. 2021 Apr;

47. Adam D. What scientists know about new, fast-spreading coronavirus variants. Nature. 2021 Jun;594(7861):19–20.

48. Tegally H, Wilkinson E, Giovanetti M, Iranzadeh A, Fonseca V, Giandhari J, et al. Emergence and rapid spread of a new severe acute respiratory syndrome-related coronavirus 2 (SARS-CoV-2) lineage with multiple spike mutations in South Africa [Internet]. Vol. 10, medRxiv. Constantinos; 2020 [cited 2021 Jun 15]. p. 2020.12.21.20248640. Available from: https://doi.org/10.1101/2020.12.21.20248640

49. Harvey WT, Carabelli AM, Jackson B, Gupta RK, Thomson EC, Harrison EM, et al. SARS-CoV-2 variants, spike mutations and immune escape. Nat Rev Microbiol. 2021 Jun;19(7):409–24.

50. 7. Hirotsu Y, Omata M. Discovery of a SARS-CoV-2 variant from the P.1 lineage harboring K417T/E484K/N501Y mutations in Kofu, Japan. Journal of Infection [Internet]. 2021 Jun 1 [cited 2021 Jun 17];82(6):276–316. Available from: https://www.journalofinfection.com/article/S0163-4453(21)00130-4/fulltext

51. Wall EC, Wu M, Harvey R, Kelly G, Warchal S, Sawyer C, et al. Neutralising antibody activity against SARS-CoV-2 VOCs B.1.617.2 and B.1.351 by BNT162b2 vaccination. The Lancet [Internet]. 2021 Jun 3 [cited 2021 Jun 17];0(0). Available from: https://www.thelancet.com/journals/lancet/article/PIIS0140-6736(21)01290-3/fulltext

52. Cherian S, Potdar V, Jadhav S, Yadav P, Gupta N, Das M, et al. Convergent evolution of SARS-CoV-2 spike mutations, L452R, E484Q and P681R, in the second wave of COVID-19 in Maharashtra, India. bioRxiv [Internet]. 2021 May 3 [cited 2021 Jun 15];2021.04.22.440932. Available from: https://doi.org/10.1101/2021.04.22.440932

53. Zhou H, Dcosta BM, Samanovic MI, Mulligan MJ, Landau NR. The Spike Proteins of SARS-CoV-2 B.1.617 and B.1.618 Variants Identified in India 1 Provide Partial Resistance to Vaccine-elicited and Therapeutic Monoclonal 2 Antibodies. 3 4 Takuya Tada. bioRxiv [Internet]. 2021 May 16 [cited 2021 Jun 15];2021.05.14.444076. Available from: https://doi.org/10.1101/2021.05.14.444076

54. Anwar S, Nasrullah M, Hosen MJ. COVID-19 and Bangladesh: Challenges and How to Address Them. Front Public Heal. 2020 Apr;8:154.

55. Kabir H, Maple M, Usher K. The impact of COVID-19 on Bangladeshi readymade garment (RMG) workers. J Public Health (Oxf). 2021 Apr;43(1):47–52.

56. Bangladesh: WHO Coronavirus Disease (COVID-19) Dashboard With Vaccination Data | WHO Coronavirus (COVID-19) Dashboard With Vaccination Data [Internet]. [cited 2021 Jun 15]. Available from: https://covid19.who.int/region/searo/country/bd

57. Saha S, Malaker R, Sajib MSI, Hasanuzzaman M, Rahman H, Ahmed ZB, et al. Complete Genome Sequence of a Novel Coronavirus (SARS-CoV-2) Isolate from Bangladesh.

Microbiol Resour Announc. 2020 Jun;9(24).

58. Moniruzzaman M, Hossain MU, Islam MN, Rahman MH, Ahmed I, Rahman TA, et al. Coding-Complete Genome Sequence of SARS-CoV-2 Isolate from Bangladesh by Sanger Sequencing. Microbiol Resour Announc. 2020 Jul;9(28).

59. Elbe S, Buckland-Merrett G. Data, disease and diplomacy: GISAID's innovative contribution to global health. Glob Challenges. 2017 Jan;1(1):33–46.

60. Wu F, Zhao S, Yu B, Chen YM, Wang W, Song ZG, et al. A new coronavirus associated with human respiratory disease in China. Nature. 2020;579(7798):265–9.

61. 5. Mercatelli D, Giorgi FM. Geographic and Genomic Distribution of SARS-CoV-2 Mutations. Frontiers in Microbiology. 2020 Jul 22;11.

62. Delcher AL, Phillippy A, Carlton J, Salzberg SL. Fast algorithms for large-scale genome alignment and comparison. Nucleic Acids Res. 2002 Jun;30(11):2478–83.

63. 4. Villanueva RAM, Chen ZJ. ggplot2: Elegant Graphics for Data Analysis (2nd ed.). Measurement: Interdisciplinary Research and Perspectives. 2019 Jul 3;17(3):160–7.

64. 3. Kim DE, Chivian D, Baker D. Protein structure prediction and analysis using the Robetta server. Nucleic Acids Research. 2004 Jul 1;32(Web Server):W526–31.

65. Park T, Baek M, Lee H, Seok C. GalaxyTongDock: Symmetric and asymmetric

ab initio protein–protein docking web server with improved energy parameters. Journal of Computational Chemistry. 2019 Jun 7;40(27):2413–7.

66. 1. Xue LC, Rodrigues JP, Kastritis PL, Bonvin AM, Vangone A. PRODIGY: a web server for predicting the binding affinity of protein–protein complexes. Bioinformatics. 2016 Aug

8.

67. Abraham MJ, Murtola T, Schulz R, Páll S, Smith JC, Hess B, et al. Gromacs: High performance molecular simulations through multi-level parallelism from laptops to supercomputers. SoftwareX. 2015 Sep 1;1–2:19–25.

68. Schuler LD, Daura X, Van Gunsteren WF. An Improved GROMOS96 Force Field for Aliphatic Hydrocarbons in the Condensed Phase. Vol. 22, Journal of Computational Chemistry. 2001.

69. Islam MT, Talukder AK, Siddiqui MN, Islam T. Tackling the COVID-19 pandemic: The Bangladesh perspective. J Public health Res. 2020;9(4):389–97.

70. Callaway E. The coronavirus is mutating - does it matter? Vol. 585, Nature. NLM (Medline); 2020. p. 174–7.

71. Vilar S, Isom DG. One year of SARS-CoV-2: How much has the virus changed? Biology (Basel). 2021 Feb;10(2):1–18.

72. Ko K, Nagashima S, Bunthen E, Ouoba S, Akita T, Sugiyama A, et al. Molecular characterization and the mutation pattern of SARS-CoV-2 during first and second wave outbreaks in Hiroshima, Japan. PLoS One. 2021 Feb;16(2 February):e0246383.

73. McCarthy KR, Rennick LJ, Nambulli S, Robinson-McCarthy LR, Bain WG, Haidar G, et al. Recurrent deletions in the SARS-CoV-2 spike glycoprotein drive antibody escape. Science (80-). 2021 Mar;371(6534):1139–42.

74. GISAID - Clade and lineage nomenclature aids in genomic epidemiology of active hCoV-19 viruses.

75. Han AX, Parker E, Scholer F, Maurer-Stroh S, Russell CA. Phylogenetic clustering by linear integer programming (PhyCLiP). Mol Biol Evol. 2019 Jul;36(7):1580–95.

76. Rambaut A, Holmes EC, O'Toole Á, Hill V, McCrone JT, Ruis C, et al. A dynamic nomenclature proposal for SARS-CoV-2 lineages to assist genomic epidemiology. Nat Microbiol. 2020 Nov 1;5(11):1403–7.

77. Tracking SARS-CoV-2 variants [Internet]. [cited 2021 Jun 15]. Available from: https://www.who.int/en/activities/tracking-SARS-CoV-2-variants/

78. Wang Q, Zhang Y, Wu L, Niu S, Song C, Zhang Z, et al. Structural and Functional Basis of SARS-CoV-2 Entry by Using Human ACE2. Cell. 2020 May;181(4):894-904.e9.

79. Arantes PR, Saha A, Palermo G. Fighting covid-19 using molecular dynamics simulations. ACS Cent Sci. 2020 Oct;6(10):1654–6.

80. Martí D, Torras J, Bertran O, Turon P, Alemán C. Temperature effect on the SARS-CoV-2: A molecular dynamics study of the spike homotrimeric glycoprotein. Comput Struct Biotechnol J. 2021 Jan;19:1848–62.

81. Sixto-López Y, Correa-Basurto J, Bello M, Landeros-Rivera B, Garzón-Tiznado JA, Montaño S. Structural insights into SARS-CoV-2 spike protein and its natural mutants found in Mexican population. Sci Rep. 2021 Dec;11(1):4659.

82. Lon JR, Xi B, Zhong B, Zheng Y, Guo P, Chen Z, et al. Molecular dynamics simulation study of effects of key mutations in SARS-CoV-2 on protein structures.

83. Wu S, Tian C, Liu P, Guo D, Zheng W, Huang X, et al. Effects of SARS-CoV-2 mutations on protein structures and intraviral protein–protein interactions. J Med Virol. 2021

Apr;93(4):2132–40.

84. Korber B, Fischer WM, Gnanakaran S, Yoon H, Theiler J, Abfalterer W, et al. Tracking Changes in SARS-CoV-2 Spike: Evidence that D614G Increases Infectivity of the COVID-19 Virus. Cell. 2020 Aug 20;182(4):812-827.e19.

Supplementary Information

Supplementary File 1. Sample_info_of_wave-1

Supplementary File 2. Sample_info_of_wave-2

Supplementary File 3. ref_annot

Supplementary File 4. full_report

Supplementary File 5. mutation_number_in_samples

Supplementary File 6. mutation_classes

Supplementary File 7. variant_types

Supplementary File 8. nucleotide_events

Supplementary File 9. protein_events

Supplementary File 10. variant_and_clades_distribution

Fig 1

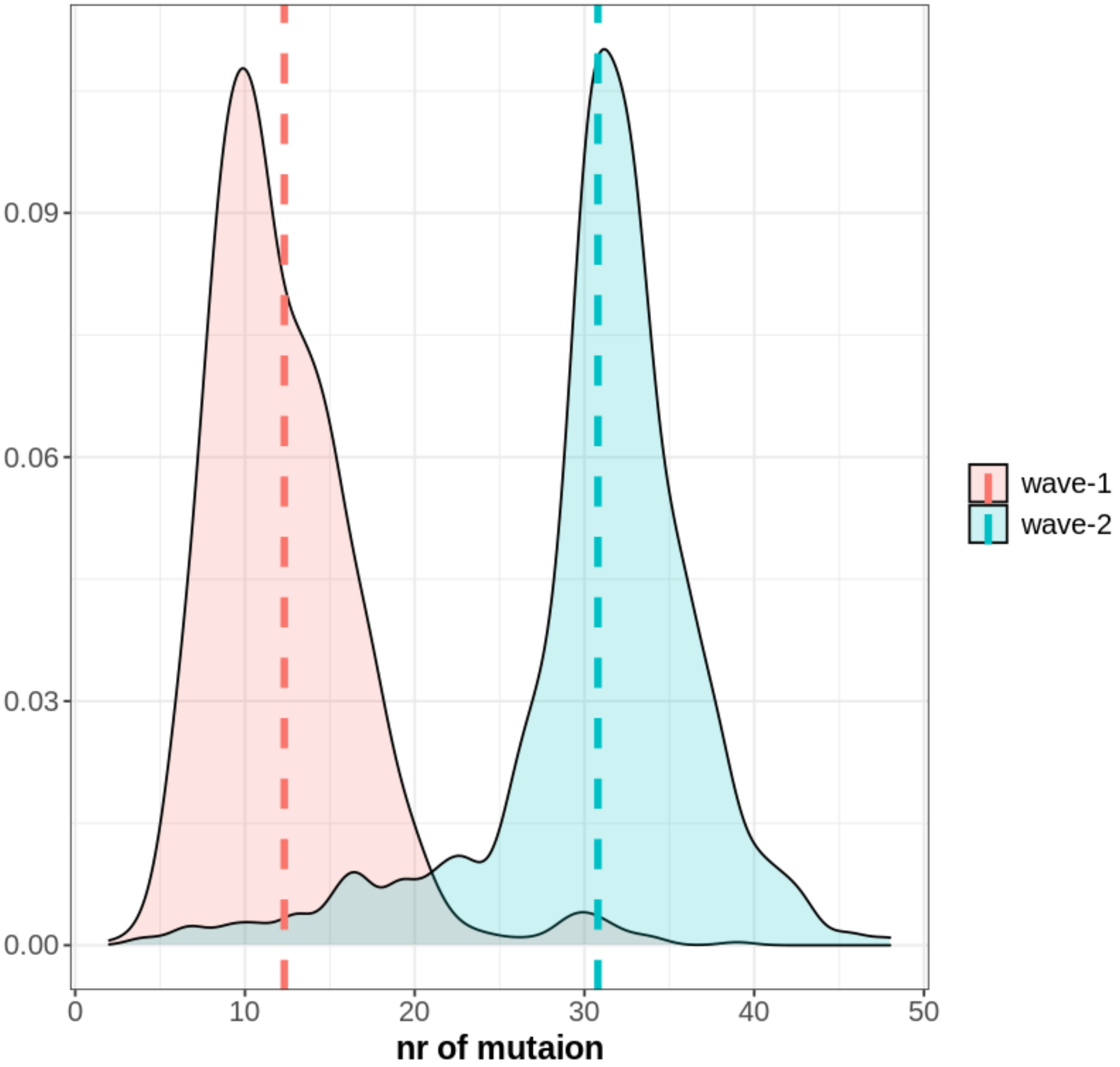

Fig 2

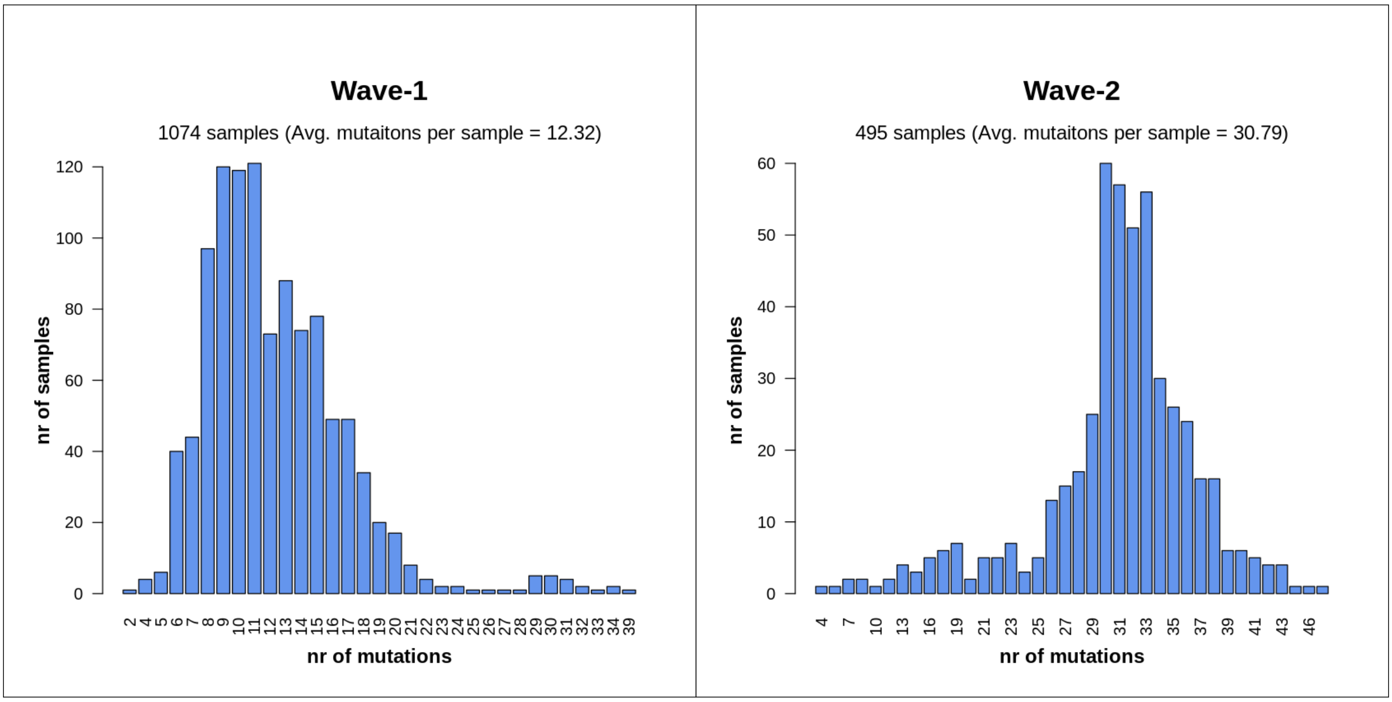
Wave-1
1074 samples (Avg. mutaitons per sample = 12.32)
nr of samples
0
20
40
60
80
100
120
2 4 5 6 7 8 9 10 11 12 13 14 15 16 17 18 19 20 21 22 23 24 25 26 27 28 29 30 31 32 33 34 39
nr of mutations
Wave-2
495 samples (Avg. mutaitons per sample = 30.79)
nr of samples
0
10
20
30
40
50
60
4 7 10 13 16 19 21 23 25 27 29 31 33 35 37 39 41 43 46
nr of mutations

Fig 3

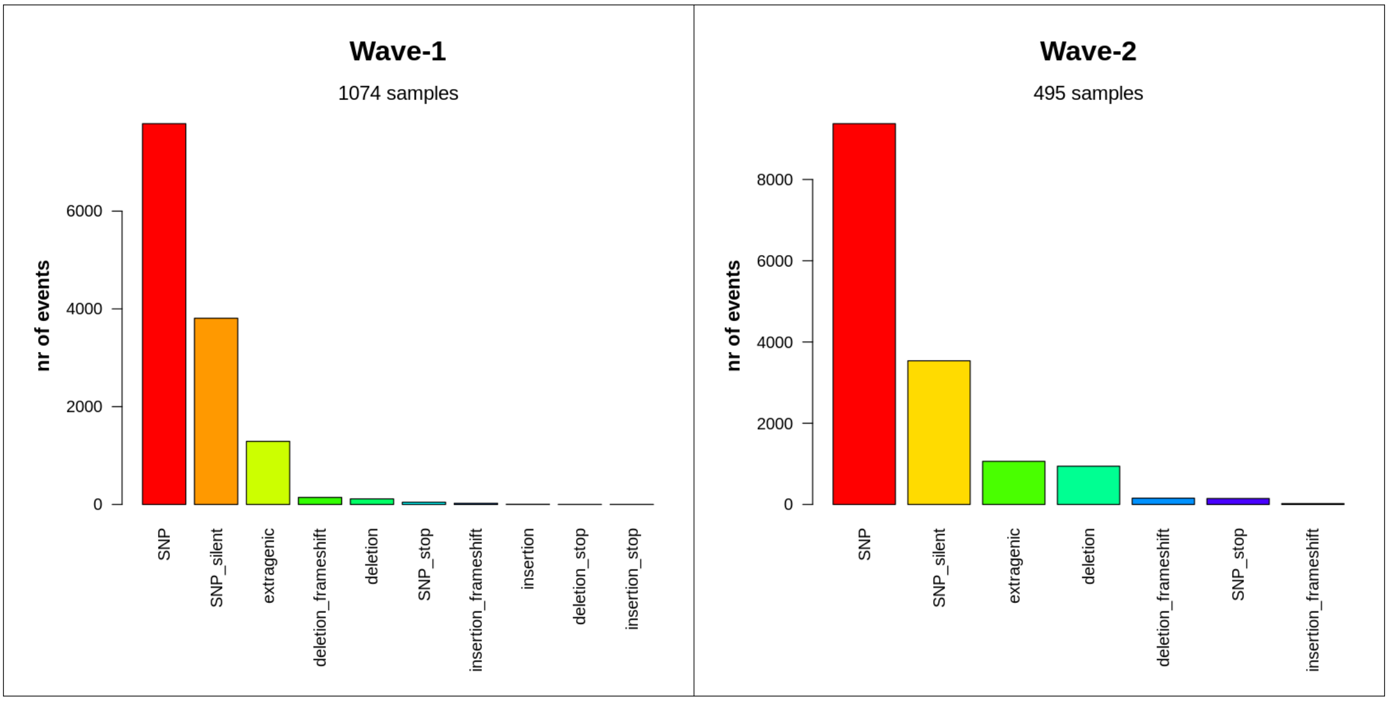
Wave-1
1074 samples
nr of events
0
2000
4000
6000
SNP
SNP_silent
extragenic
deletion_frameshift
deletion
SNP_stop
insertion_frameshift
insertion
deletion_stop
insertion_stop
Wave-2
495 samples
nr of events
0
2000
4000
6000
8000
SNP
SNP_silent
extragenic
deletion
deletion_frameshift
SNP_stop
insertion_frameshift

Fig 4

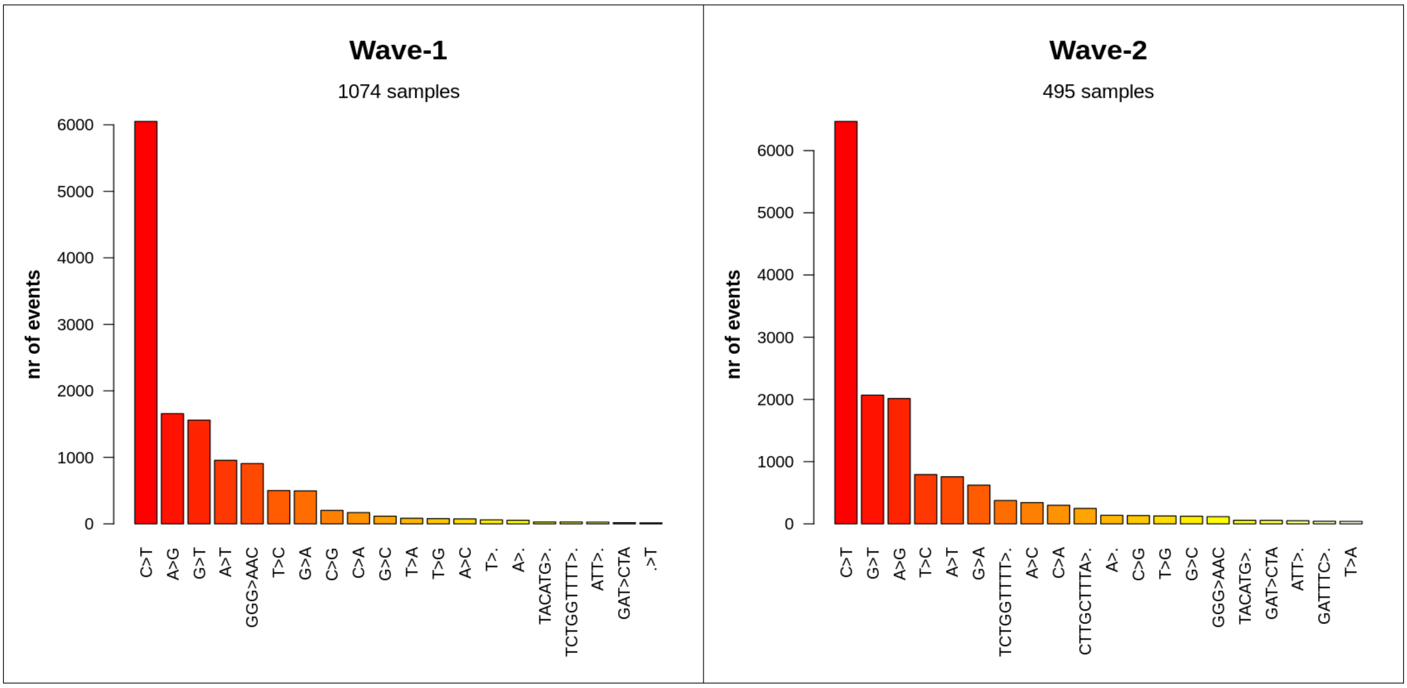
Wave-1
1074 samples
nr of events
6000
5000
4000
3000
2000
1000
0
C>T
A>G
G>T
A>T
GGG>AAC
T>C
G>A
C>G
C>A
G>C
T>A
T>G
A>C
T>.
A>.
TACATG>.
TCTGGTTTT>.
ATT>.
GAT>CTA
.>T
Wave-2
495 samples
nr of events
6000
5000
4000
3000
2000
1000
0
C>T
G>T
A>G
T>C
A>T
G>A
TCTGGTTTT>.
A>C
C>A
CTTGCTTTA>.
A>.
C>G
T>G
G>C
GGG>AAC
TACATG>.
GAT>CTA
ATT>.
GATTTC>.
T>A

Fig 5

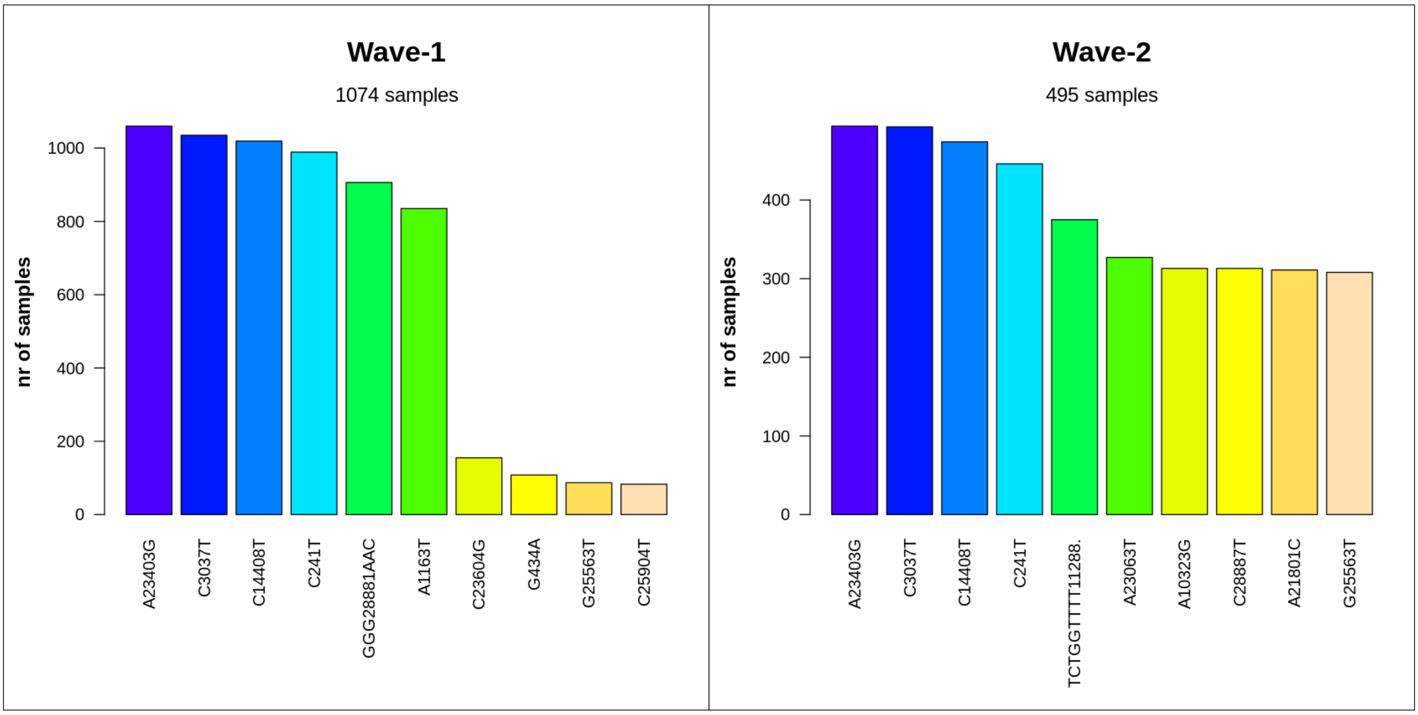
Wave-1
1074 samples
nr of samples
1000
800
600
400
200
0
A23403G
C3037T
C14408T
C241T
GGG28881AAC
A1163T
C23604G
G434A
G25563T
C25904T
Wave-2
495 samples
nr of samples
400
300
200
100
0
A23403G
C3037T
C14408T
C241T
TCTGGTTTT11288.
A23063T
A10323G
C28887T
A21801C
G25563T

Fig 6

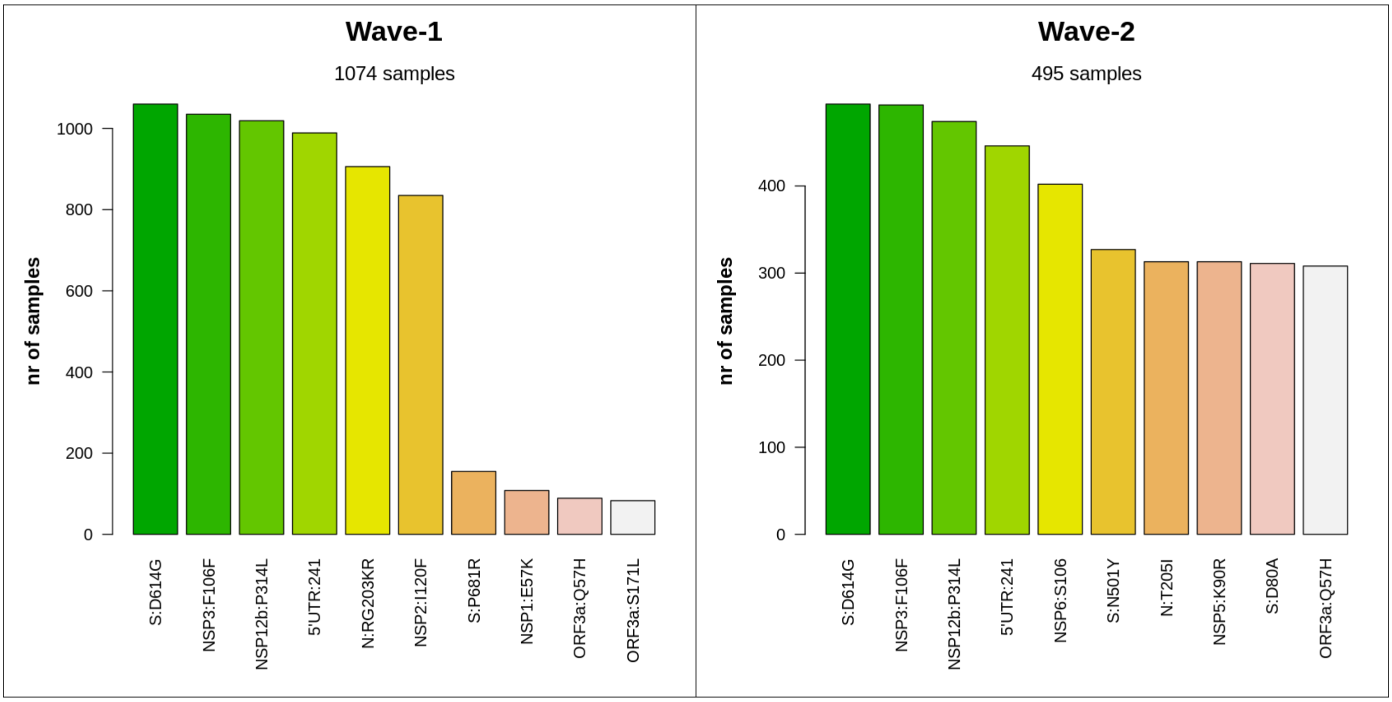
Wave-1
1074 samples
nr of samples
0
200
400
600
800
1000
S:D614G
NSP3:F106F
NSP12b:P314L
5'UTR:241
N:RG203KR
NSP2:I120F
S:P681R
NSP1:E57K
ORF3a:Q57H
ORF3a:S171L
Wave-2
495 samples
nr of samples
0
100
200
300
400
S:D614G
NSP3:F106F
NSP12b:P314L
5'UTR:241
NSP6:S106
S:N501Y
N:T205I
NSP5:K90R
S:D80A
ORF3a:Q57H

Fig 7

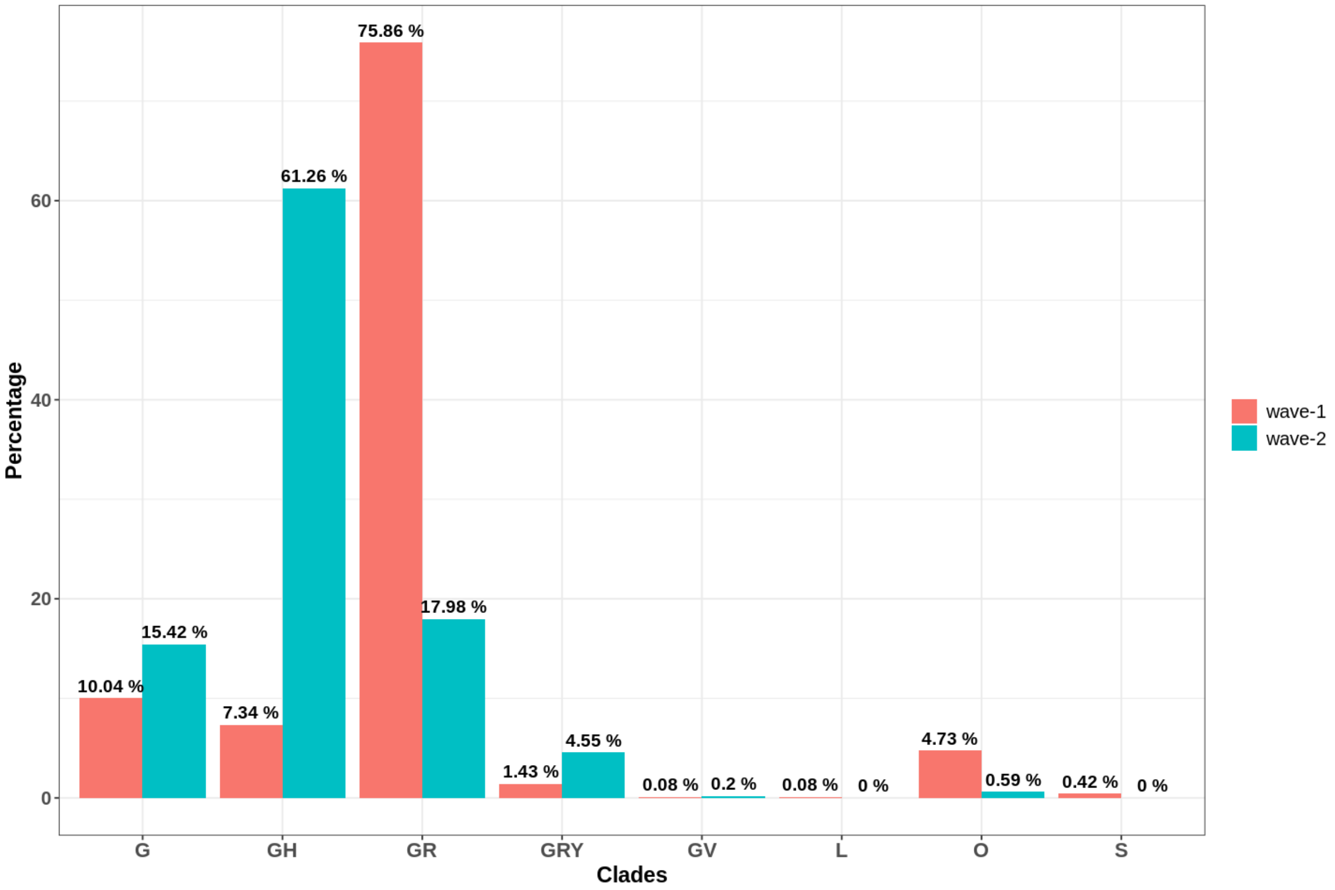
75.86 %
61.26 %
17.98 %
15.42 %
10.04 %
7.34 %
4.55 %
1.43 %
0.08 %
0.2 %
0.08 %
0 %
4.73 %
0.59 %
0.42 %
0 %
Percentage
60
40
20
0
G
GH
GR
GRY
GV
L
O
S
Clades
wave-1
wave-2

Fig 8

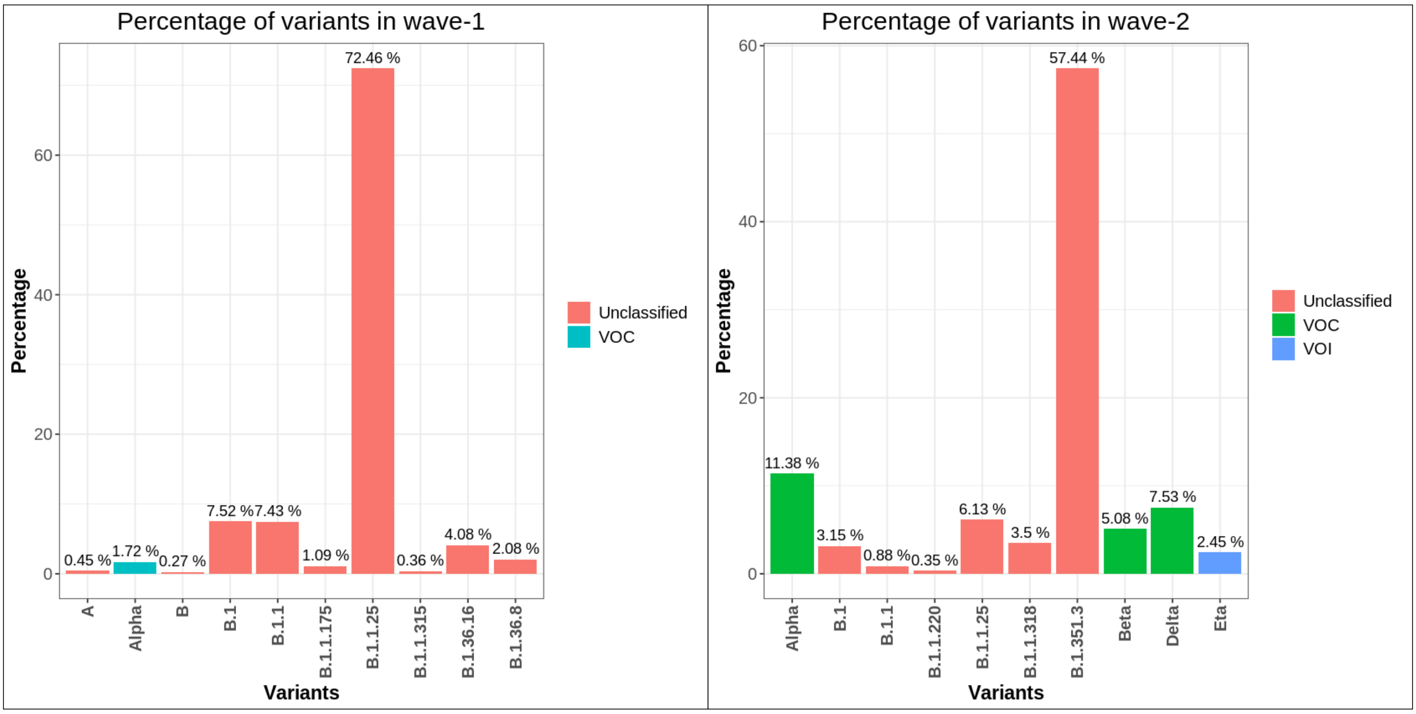
Percentage of variants in wave-1
Percentage
Variants
0
20
40
60
72.46 %
0.45 %
1.72 %
0.27 %
7.52 %
7.43 %
1.09 %
0.36 %
4.08 %
2.08 %
A
Alpha
B
B.1
B.1.1
B.1.1.175
B.1.1.25
B.1.1.315
B.1.36.16
B.1.36.8
Unclassified
VOC
Percentage of variants in wave-2
Percentage
Variants
0
20
40
60
57.44 %
11.38 %
3.15 %
0.88 %
0.35 %
6.13 %
3.5 %
5.08 %
7.53 %
2.45 %
Alpha
B.1
B.1.1
B.1.1.220
B.1.1.25
B.1.1.318
B.1.351.3
Beta
Delta
Eta
Unclassified
VOC
VOI

Fig 9a

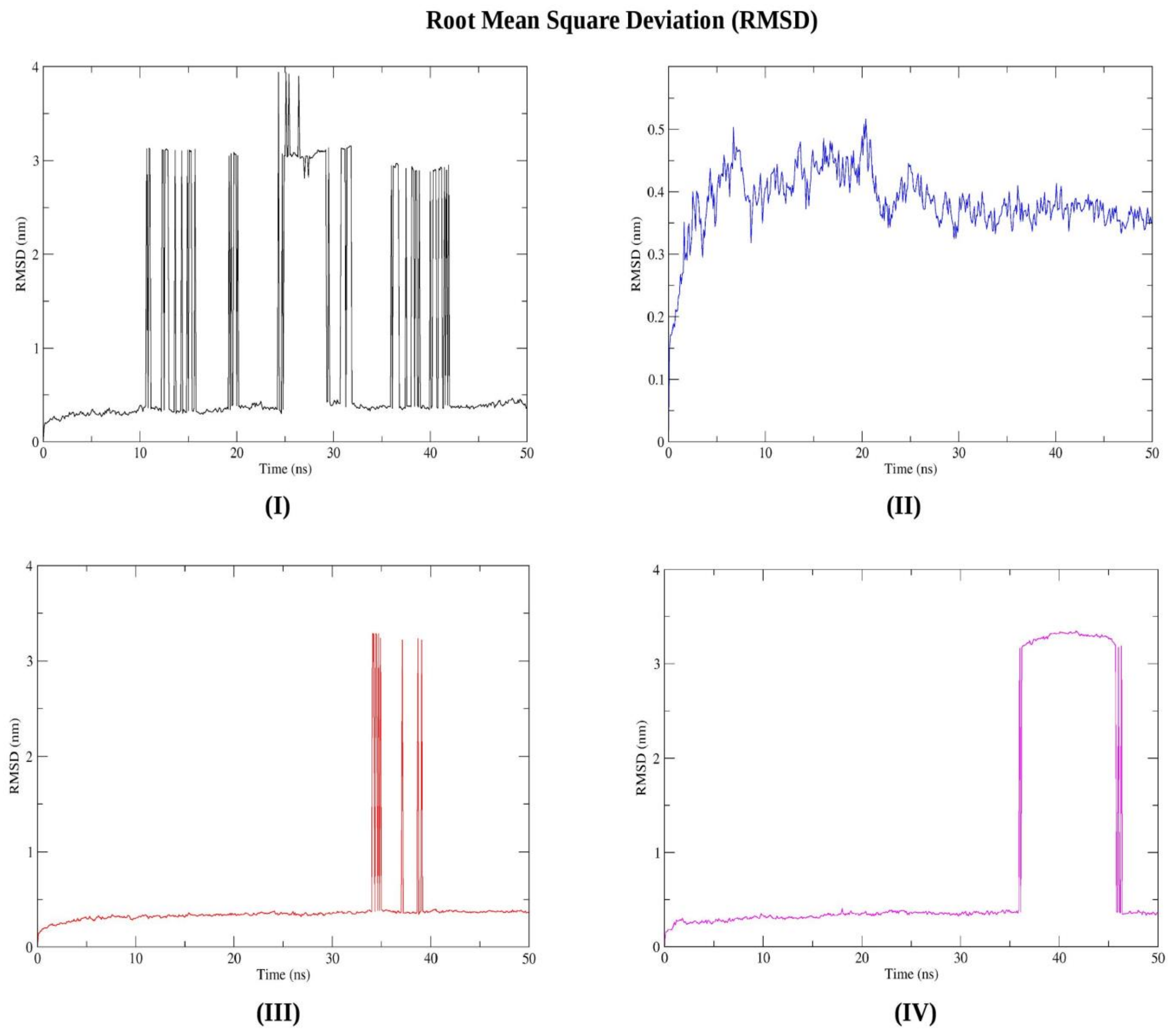

Fig 9b

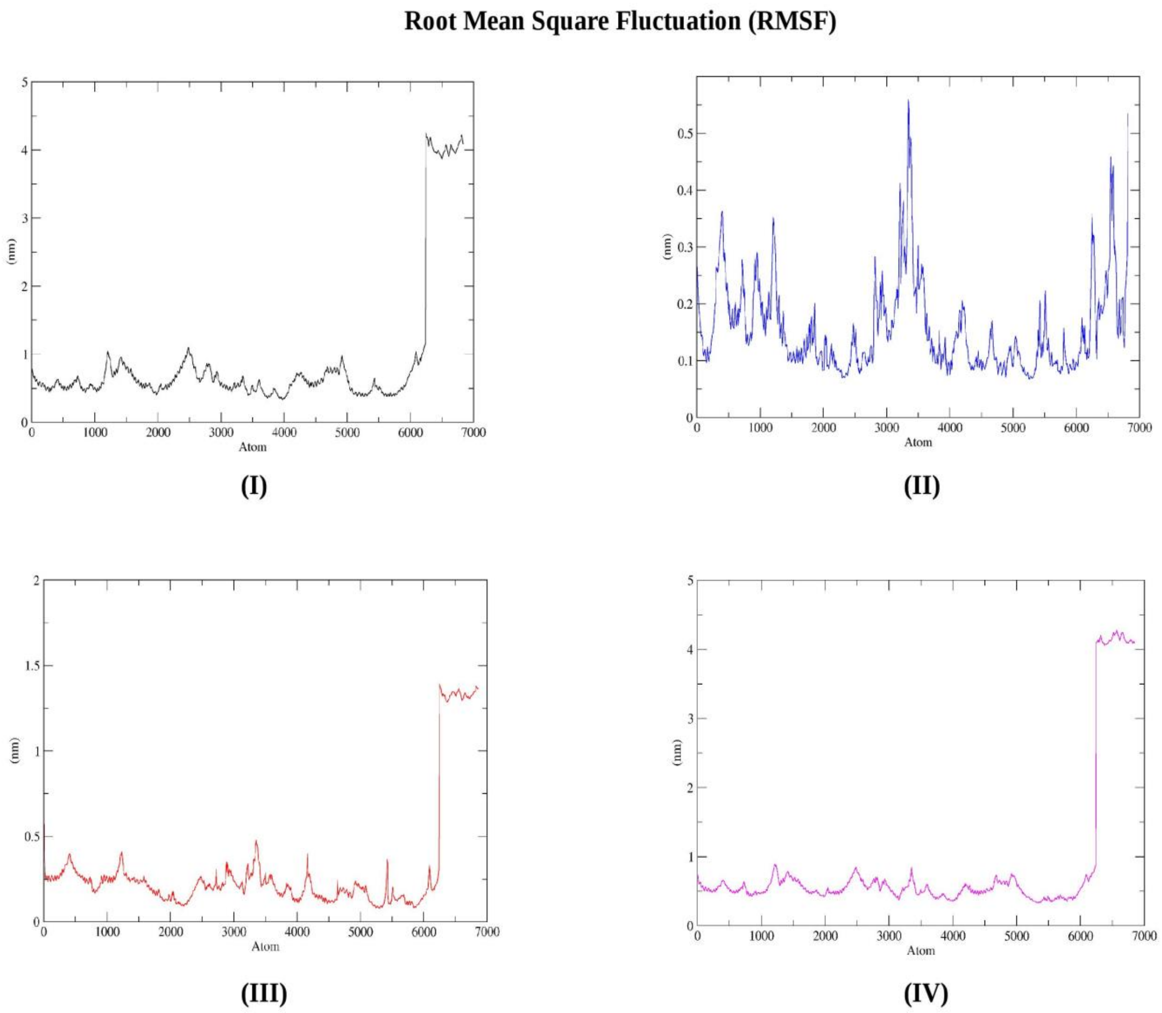

.

Fig 9c

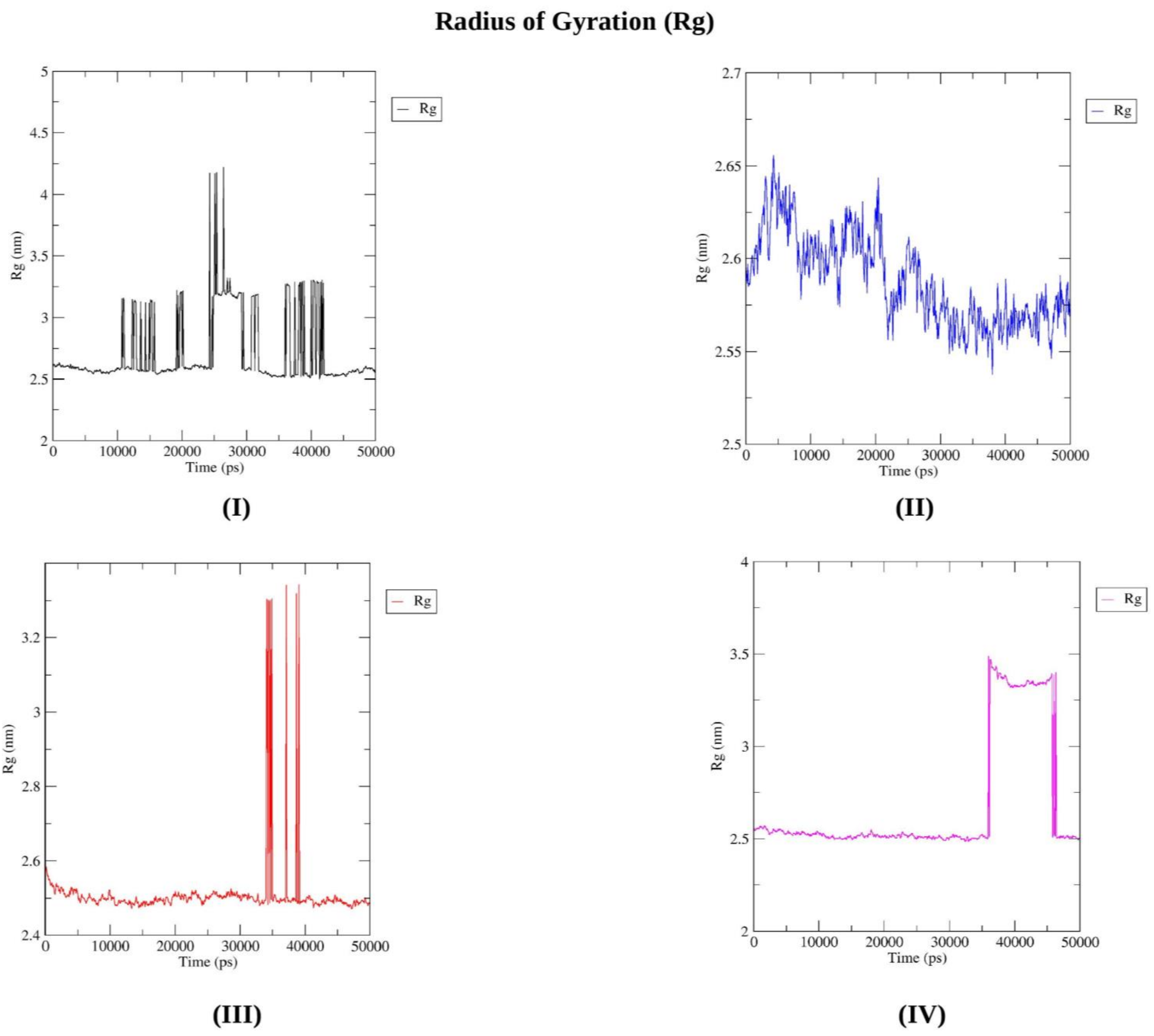

Fig 9d

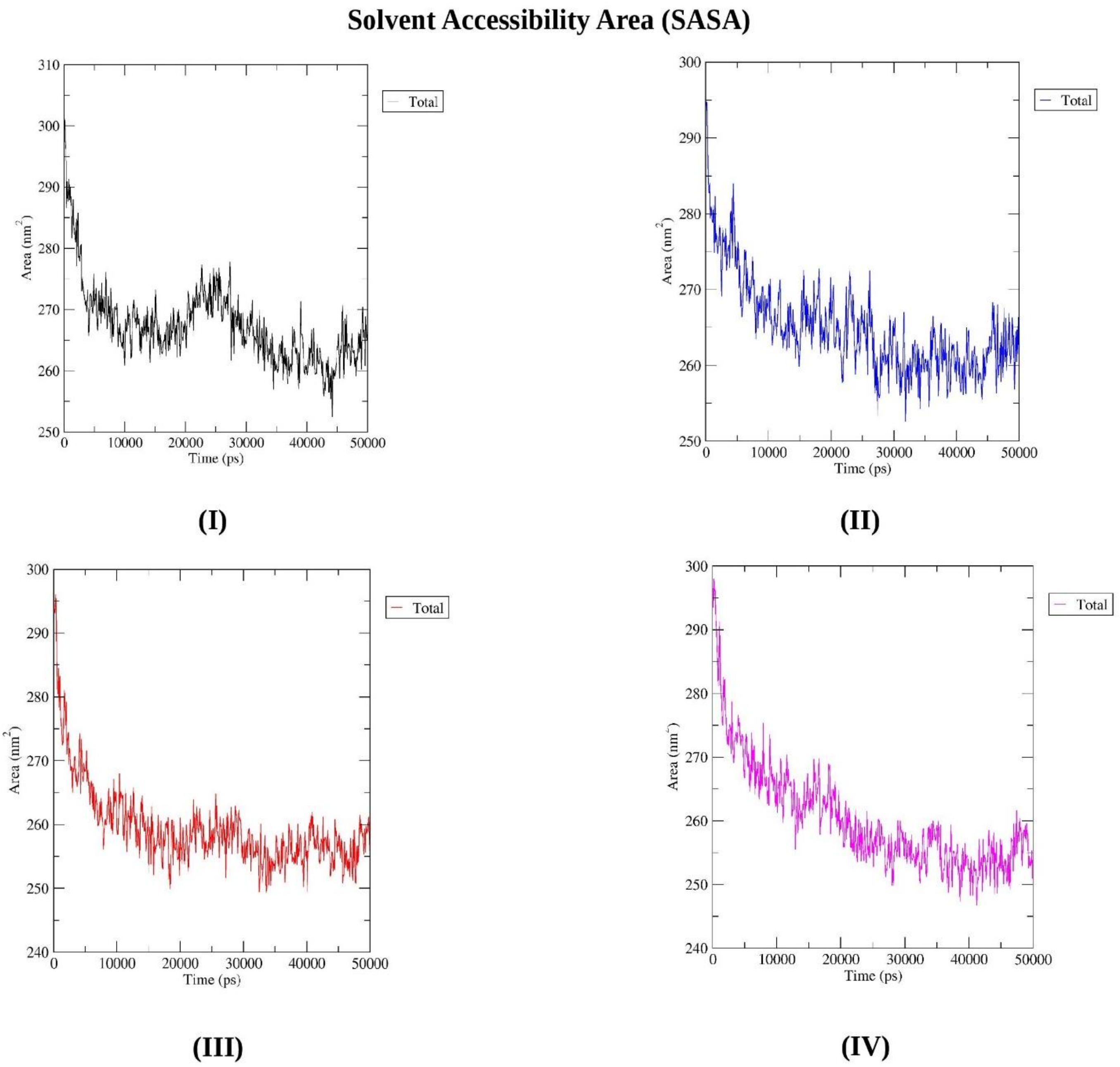
Solvent Accessibility Area (SASA)
Area (nm^2)
Time (ps)
Total
(I)
(II)
(III)
(IV)

Click here to access/download

Supporting Information - Compressed/ZIP File Archive

Supplementary_files.zip
