# Supplementary material for: Wave-wise comparative genomic study for revealing the complete scenario and dynamic nature of COVID-19 pandemic in Bangladesh
Source: PLoS One. 2021 Sep 29;16(9):e0258019. doi: 10.1371/journal.pone.0258019 (PMC8480844; doi:10.1371/journal.pone.0258019)
Supplement: Attachment — Submitted filename: Response to Reviewers.docx [file pone.0258019.s014.docx]

\# Response to Queries from Reviewer 1

**Reviewer’s Query:**

The manuscript “Comparative Genomic Study for Revealing the Complete Scenario of COVID-19 Pandemic in Bangladesh” (PONE-D-21-19985) reports the SARS-CoV2 variants that have caused first and second wave in Bangladesh. The authors have compared the causative variants responsible for the two waves. The manuscript seems to be the first report detailing the molecular perspectives of the two consecutive waves of COVID19 infection in Bangladesh. The evolutionary perspectives of the viral genome has been studied. However, there are some queries which must be addressed by the authors to improve the manuscript.

Abstract: OK.

Introduction:

Page 8; Line: 31-32: Please clearly state based on analyses of which molecular sequences you are stating that “it was discovered that the wave-2 samples had a significantly greater average rate of mutation/sample (30.79%) than the wave-1 samples (12.32%)”

**Authors’ Response**

Abstract/Introduction

**1.** Information about the type of molecular sequences has been added into the abstract which can be found at **page 2, line 30-33**.

**Reviewer’s Query:**

Materials and Methods:

Page 16, Line 208: Please correct “Room Mean Square Fluctuation (RMSF)” to “Root Mean Square Fluctuation (RMSF)”.

**Authors’ Response**

Materials and Methods

“Room Mean Square Fluctuation (RMSF)” has been changed to “Root Mean Square Fluctuation (RMSF)” at **page 10, line 222**.

**Reviewer’s Query:**

Results and Discussion:

1. Please specify the length of genomes that have been studied

2. What was the quality of sequencing (base calling)?

3. Compare the genomic constitution of the two waves or other specific variants.

4. The Spike protein is variable and have been reported to experience selection pressure (doi: 10.1016/j.heliyon.2020.e05001; DOI: 10.3390/pathogens9100829). Please report in the light of the samples of Bangladesh.

5. Please include elaborated analysis on selection pressure and the molecular phylogeny of the variants for the genes showing variation among the viral variants.

English: The formation of sentences is not correct. There are several grammatical and syntactical errors. The English of the manuscript must be get thoroughly checked by a suitable English language professional. Some of the points have been indicated in the attached manuscript.

Please don’t use the symbols like “>”, in running text, rather write “more than” or “greater than”

**Authors’ Response**

Result and Discussion

1. Length of the genomes has been added at **page 11, line 241-243.**

2. The study was performed on assembled genomes from GISAID database. GISAID does not contain raw reads or quality score of the sequences. However, we only chose complete genomes for our study.

3. Comparison of genomic constitution between wave-1 and wave-2 has been provided in the following paragraphs;

3.2 Frequency of SARS-CoV-2 Mutations in Bangladesh

3.3 Type of SARS-CoV-2 Mutations in Bangladesh

3.4 Genomic Location of the SARS-CoV-2 Mutations

3.6 Distribution of SARS-CoV-2 Clades and Variants in Bangladesh

4 and 5. Analysis of selection pressure and changes in the variability in spike protein have been calculated and discussed along with molecular phylogeny of the variants. The newly added parts in this regard can be found in the following sections of the manuscript-

Material methods section

2.4 Analysis on Selection Pressure and Molecular Phylogeny of The Variants in **page 8-9**, **line 173-180.**

Result section

3.7 Selection Pressure and Phylogenetic Analysis in **page 16-17,** **line 339-355.**

Discussion

The findings regarding selection pressure and phylogeny analysis are discussed **in page 23-24, line 468 to 483.**

Figure and Supplementary file

Newly added Figures and Supplementary files-

**Fig 9** in **page 47, line 815**

**Supplementary file 11**

English:

- “>” sign previously used to denote mutations has been replaced with “to” in running text.
- Grammatical adjustments as highlighted in the honorable reviewer's attachment has been carried out. The whole of the manuscript was also checked for grammatical errors and corrected wherever needed.

\# Response to Queries from Reviewer 2

**Reviewer’s Query:**

The manuscript presented falls within the scope of the Journal and adds knowledge about the distribution of various clades of SARS-CoV-2 in Bangladesh. The manuscript is well written with enough data both in terms of extensiveness and representation.

However there are certain minor corrections in the manuscript which can be found in pop-up notes of the attachment. I also suggest you to modify the title as indicated in the pop-up note of the title.

**Authors’ Response**

We thank the honorable reviewer #2 for his suggestions for the title, considered it seriously and felt the need to modify the title slightly to better represent the contents of the study. The new title is as following- “Wave-wise comparative genomic study for revealing the complete scenario and dynamic nature of COVID-19 pandemic in Bangladesh”. We did not include the word “clade-wise” in the title because the study was not just based on distribution of various clades of SARS-CoV-2 in Bangladesh but a complete scenario of the pandemic from a comparative genomics perspective. For example, we analyzed the distribution of mutations per sample, classes of mutations, most frequent mutational events, distribution of different clades, prevalence of different variants, selection pressure and phylogenetic analysis between wave-1 and wave-2 as well as the effect of mutations on the transmissibility of the virus. Honorable reviewer #2 also suggested to include the phrase “Genomic Surveillance” in the title. Even though our study encompassed genomic surveillance, it was more than that. Our main focus was on the comparison of genomic features of SARS-CoV-2 between the first and the second wave in Bangladesh. Therefore, we used “Wave-wise comparative genomic study” instead of “Genomic Surveillance” in the title to denote the nature of our work.

Other minor corrections suggested by honorable reviewer #2 has also been executed in the revised manuscript.
